# Fungal lifestyle reflected in serine protease repertoire

**DOI:** 10.1038/s41598-017-09644-w

**Published:** 2017-08-22

**Authors:** Anna Muszewska, Marta M. Stepniewska-Dziubinska, Kamil Steczkiewicz, Julia Pawlowska, Agata Dziedzic, Krzysztof Ginalski

**Affiliations:** 1 Institute of Biochemistry and Biophysics, Polish Academy of Sciences, Pawinskiego 5A, 02-106 Warsaw, Poland; 2 Laboratory of Bioinformatics and Systems Biology, Centre of New Technologies, University of Warsaw, Zwirki i Wigury 93, 02-089 Warsaw, Poland; 3 Department of Molecular Phylogenetics and Evolution, Faculty of Biology, Biological and Chemical Research Centre, University of Warsaw, Zwirki i Wigury 101, 02-089 Warsaw, Poland

## Abstract

Fungi are able to switch between different lifestyles in order to adapt to environmental changes. Their ecological strategy is connected to their secretome as fungi obtain nutrients by secreting hydrolytic enzymes to their surrounding and acquiring the digested molecules. We focus on fungal serine proteases (SPs), the phylogenetic distribution of which is barely described so far. In order to collect a complete set of fungal proteases, we searched over 600 fungal proteomes. Obtained results suggest that serine proteases are more ubiquitous than expected. From 54 SP families described in MEROPS Peptidase Database, 21 are present in fungi. Interestingly, 14 of them are also present in Metazoa and Viridiplantae – this suggests that, except one (S64), all fungal SP families evolved before plants and fungi diverged. Most representatives of sequenced eukaryotic lineages encode a set of 13–16 SP families. The number of SPs from each family varies among the analysed taxa. The most abundant are S8 proteases. In order to verify hypotheses linking lifestyle and expansions of particular SP, we performed statistical analyses and revealed previously undescribed associations. Here, we present a comprehensive evolutionary history of fungal SP families in the context of fungal ecology and fungal tree of life.

## Introduction

Serine proteases (SPs) are essential hydrolytic enzymes that utilize the catalytic serine residue for cleaving peptide bonds in proteins^1^. They can be found in all living organisms and perform a variety of functions ranging from housekeeping: e.g. protein maturation, signal peptide cleavage, signal transduction, intracellular protein turnover, immune response, apoptosis, reproduction^2^ and cytochrome processing in mitochondria, to nutrient breakdown and acquisition. These diverse roles require proteases with varied specificities, ranging from rather unspecific digestive proteases that cleave after hydrophobic or positively charged residues, to more specialized proteases that recognize a well-defined motif or even a particular protein^2^. SPs constitute a key component of the degradome of all organisms^1^. They are also used in industry as detergents and in molecular biology as protein degrading agents, e.g. during nucleic acid purification^3^. MEROPS database^4^, which stores current systematic classification of all known peptidases, groups 52 SP families into 15 clans: 12 exclusively of serine- and 3 of mixed-catalytic type (spanning cysteine, serine and threonine proteases)^4^. Most of them use the aspartate-histidine-serine (DHS) catalytic triad, which, despite a preserved common spatial arrangement, can be found in proteins classified to distinct structural folds. The catalytic mechanism involving the DHS triad emerged independently several times and is a well-studied example of convergent evolution^3, 5^.

Fungi are heterotrophic eukaryotes producing hyphae with a cell wall composed most commonly of chitin and glucans. They employ various life strategies ranging from obligate biotrophic mutualists and pathogens to specialized wood decomposers and opportunistic saprobes. Fungi like other Eukaryotes possess an extensive set of housekeeping, intracellular SPs involved in protein turnover, protein maturation, signal transduction and signal peptide cleavage to mention only a few, as well as a range of secreted SPs. Since fungi are sessile osmotrophic organisms, their lifestyle is reflected in the repertoire of secreted enzymes, which play a pivotal roles in nutrient degradation and subsequent assimilation, and protection from host’s immune system. Specifically, SPs are involved in host-fungus interactions either pathogenic or symbiotic^6^. For instance, serine proteases can be used to escape the host’s immune system by degrading chitinases that target fungal cell wall^7^. Fungal SPs are also crucial for nutrient acquisition from protein-rich sources of both plant and animal origin. They have been described for entomophatogenic^8^, nematophagous^9, 10^, mycoparasitic^11^, dermatophytic^9^, plant pathogenic^12^ and endophytic^6^ fungi. Most studies of fungal SPs were focused on animal fungal pathogens and confirmed the extracellular proteolytic activity performed by SPs in *Aspergillus fumigatus* ^13^,*Candida albicans* ^14^, *Cryptococcus meningitis* ^15^, *Histoplasma capsulatum* ^16^ and *Paracoccidioides brasiliensis* ^13, 17^. Some animal-related fungi have been reported to encode more than 15 copies of secreted serine proteases from proteinase K subfamily (subtilisin, S8A) (*Metarhizium anisopliae* ^8^, *Coccidioides immitis* ^18^).

Fungal non-secreted serine proteases are involved in a variety of intracellular processes including signal peptide processing and vacuole maintenance^19–22^; they display chaperone activity and take part in recycling of other peptides. Most non-secreted SP families present in fungi have representatives also in Eukaryotes, and some are present even in bacteria^1^. Fungal intracellular SPs have been characterized mostly in *Saccharomyces cerevisiae* (for example, the mitochondrial Lon ATPase dependent protease PIM1 from S16 family^23^) and rarely in other non-model taxa. In consequence, their detailed function often remains either extrapolated from very distant organisms (e.g. *E. coli* SppA protease from S49 family degrades signal peptides in the membrane^24^) or understudied (lysosomal Pro-Xaa carboxypeptidase, S28; X-Pro dipeptidyl-peptidase, S15).

Previous studies concerning fungal repertoire of serine peptidases focused on single SPs families, e.g. trypsins, chymotrypsins and subtilases, especially those which are secreted and may play roles in pathogenesis^5, 25^. For instance, subtilases were examined as extracellular degrading enzymes secreted by many pathogens^8–12^ and as a prohormone-processing enzymes (Kex2)^26^. Another example are caseinolytic proteases (ClpP, S14), which are conserved across the tree of life and are used for intracellular protein degradation^23, 27^. Eventually, there were limited attempts to correlate serine protease repertoire with fungal ecology, which led to the assumption that trypsins (S1) may be linked to pathogenicity against plant hosts^28^ and subtilisins (S8) could be related to a saprotrophic lifestyle^25^. Noteworthy, the aforementioned studies were limited by available genomic resources which now are significantly enriched in sequences from taxa representing diverse lifestyles, and less biased towards pathogenic fungi.

In this study, by using protein sequence homology detection methods, we identified and classified all serine proteases in completely sequenced representatives of the fungal kingdom. Subsequently, we compared the obtained taxonomic distribution of SP families with the fungal taxonomy and hypothesized about the possible evolutionary scenarios leading to the observed SP abundance patterns. Our study, based on extensive sequence data, revealed previously unreported protease subfamilies, the ubiquity of housekeeping SPs in early branching fungi, and possible horizontal gene transfer (HGT) events. Moreover, we show that protease expansions are parallel to proteome size increase and are often correlated with associations with a plant host.

## Results

### The Data Set

In order to identify all SPs in fungi, we carried out iterative and exhaustive searches with representatives from each of 52 MEROPS serine protease families against 634 fungal proteomes (Supplementary Table 1a) and additional 20 proteomes of other model eukaryotes to gain the outgroups for subsequent phylogenetic inference and the context for formulation of evolutionary scenario. According to the taxonomic distribution derived straight from MEROPS database (as of October 2016), 22 SP families are present in fungi (Supplementary Table 1b). Our extensive searches yielded 23 serine protease families in fungal proteomes. However, members of two families, D-Ala-D-Ala carboxypeptidase A and C (S11 and S13, respectively), are present solely in the proteome of *Cordyceps bassiana* D1–5, but are missing both in *C. bassiana* isolate ARSEF 2860 and other Corydypitaceae, and show an intriguely high similarity to *Escherichia coli* sequences which is likely a result of contamination (other sequences from this assembly were also similar to *E. coli*) rather than an authentic occurrence. In consequence, we state that fungal SP repertoire spans 21 families only (Table 1).

**Table 1:** Summary of SP families present in fungi.

MEROPS ID	MEROPS name	Common names	Functions	MT	Secreted	Active site residues
S1	chymotrypsin	trypsin, chymotrypsin, Nma111 peptidase, CHY1 peptidase	chaperone^72^, extracellular degradation^10^	−	+	HDS
S8	subtilisin	subtilisin, oryzin, pyrolisin, TPPII, osf, proteinase K, furin, kexin, cuticle-degrading peptidase, peptidase T	extracellular degradation^10^, intracellular pro-hormone activation^26^, intracellular protein degradation^21^	−	++	DHS
S9	prolyl oligopeptidase	prolyl oligopeptidase, dipeptidyl aminopeptidase A, B, oligopeptidase B, dipeptidyl-peptidase 4,5	protein maturation, alpha factor maturation^73^, extracellular degradation^10^, vacuole protease^20^	−	+	SDH
S10	carboxypeptidase Y	carboxypeptidase Y, kex carboxypeptidase, carboxypeptidase OcpA, OcpB, carboxypeptidase O	vacuole protease^19^, extracellular degradation^10^	−	++	SDH
S12	D-Ala-D-Ala carboxypeptidase B	D-Ala-D-Ala carboxypeptidase B, aminopeptidase DmpB	chitinase degradation^7^	−	+	SKY
S14	ClpP endopeptidase	Clp protease, ClpX	mitochondrial protein involved in protein maturation and stress reaction^74^	+	−	SHD
S15	X-Pro dipeptidyl-peptidase	PepX, PepXP, X-prolyl dipeptidyl aminopeptidase, X-Pro dipeptidyl-peptidase	unknown in fungi	−	−	SDH
S16	Lon	Lon protease	missfolded protein degradation in mitochondria^75^	+	+	
S24	LexA		unknown in fungi	+	−	SK
S26	signal peptidase I	mitochondrial inner membrane peptidase 1, 2, signalase	maturation of mitochondrial proteins^76^	+	++	SK
S28	lysosomal Pro-Xaa carboxypeptidase	acid prolyl endopeptidase	unknown in fungi	−	++	SDH
S33	prolyl aminopeptidase	prolyl aminopeptidase, proline protease	understudied in fungi, yeast proteins similar to proline proteases are not proteases	−	−	SDH
S41	C-terminal processing peptidase	interphotoreceptor retinoid-binding protein	caspase- and legumain-like activities^32^	−	++	SK
S45	penicillin G acylase precursor	penicillin G acylase precursor	unknown in fungi	+	++	S
S49	protease IV	protease IV, signal peptide peptidase A	unknown in fungi	−	−	KSS/SSK
S51	dipeptidase E	cyanophycinase, alpha-aspartyl dipeptidase	unknown in fungi	−	++	SHE
S53	sedolisin	aorsin, grifolisin, tripeptidyl-peptidase I	TppI – lysosomal enzyme, degradation of extracellular proteins^77^	−	++	EDDS
S54	Rhomboid	Rhomboid	mitochondrial endopeptidase^78^	+	+	SH
S59	nucleoporin 145	nucleoporin, Nup189	essential for nuclear pore formation^79^	−	+	HxS
S64	Ssy5	Ssy5	detection of sources of amino acids^41^	−	−	HDS
S66	LD-carboxylpeptidase	LD-carboxylpeptidase, murein tetrapeptidase LD-carboxypeptidase	unknown in fungi	+	−	SDH

Function assignment was based on SGD^71^ functional annotation and literature searches. MT stands for mitochondrial localization; Secreted: “+”, more than 10% predicted to be secreted; “++”, more than 50% predicted to be secreted.

The 21 SP families span 28,974 fungal serine proteases, after dismissing fragmented and likely inactive homologs (for accession identifiers see Supplementary File 1; protease counts are summarized in Supplementary Table 1d and e for fungi and outgroup model eukaryotes, respectively). Sequence collections derived for each of the 21 SP families were merged together with sequences representing a manually curated set of the corresponding Pfam families to recognize relationships between Pfam protein domain definition and MEROPS families in subsequent analyses (see Materials and Methods and Supplementary Table 1c).

### Fungi and LECA

**Figure 1:**
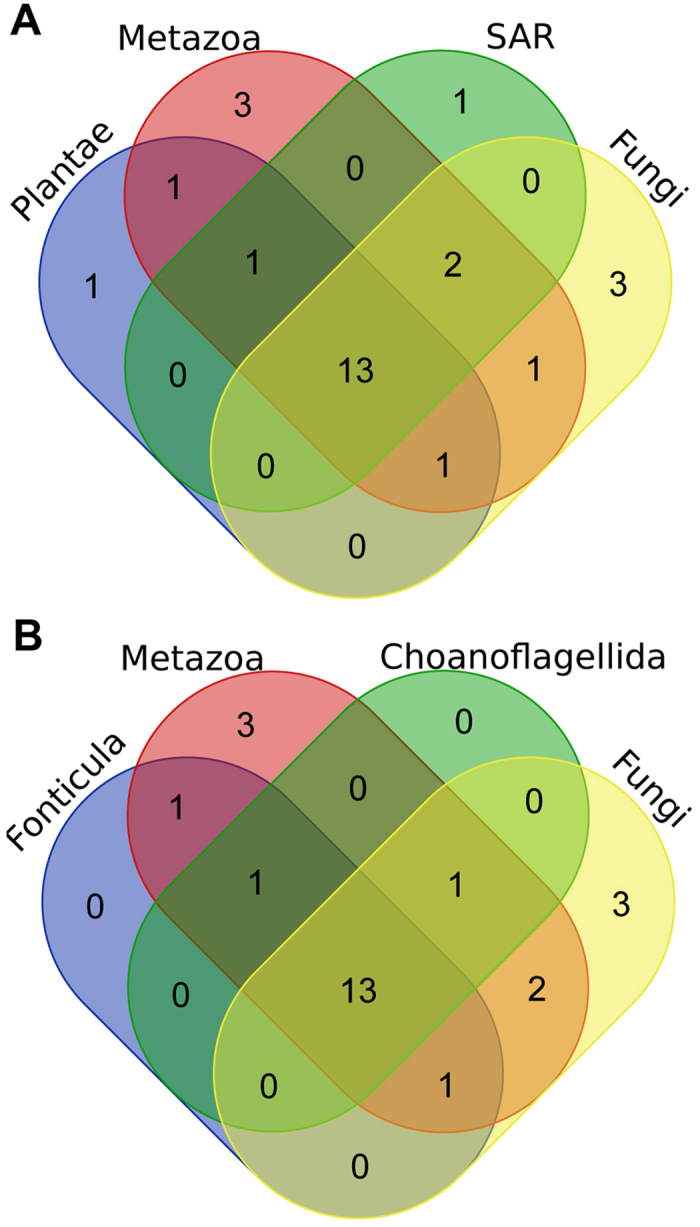
A Venn diagram representing the numbers of SP families shared in selected lineages of Eukaryota (**A**) and in main lineages of Opistokonta (**B**). The image was prepared using Draw Venn Diagram – Ugent^69^.

The major lineages of eukaryotes: plants, animals, SAR (stramenopiles, alveolates, and Rhizaria) and fungi share 13 serine proteases (S1, S8, S9, S10, S12, S14, S16, S26, S28, S33, S53, S54 and S59) which might constitute a minimal set of SPs in LECA (last eukaryotic common ancestor, see Fig. 1A). Except for plants, all major lineages retain also protease IV (S49), which extends the minimal core to 14 SP families. This scenario of common ancestry of at least 10–11 SP is even more likely as 11 and 10 out of those families are present in Bacteria and Archaea, respectively. The fungal repertoire of the most conserved SPs seems to be relatively narrow and possibly similar to the eukaryotic core. In general, fungi possess at least 6 SPs (S8, S9, S10, S16, S26 and S54) and most of the analyzed proteomes contain representatives of further six SP families (S1, S12, S14, S28, S53 and S59). Analysed Ophistokonta share 13 SP families (see Fig. 1B) and this is the lower bound of SP distribution in non-fungal organisms. LexA (S24), Ssy5 (S64) and LD-carboxylpeptidases (S66) proteases are present only in few fungal representatives.

### Basal fungal lineages

**Figure 2:**
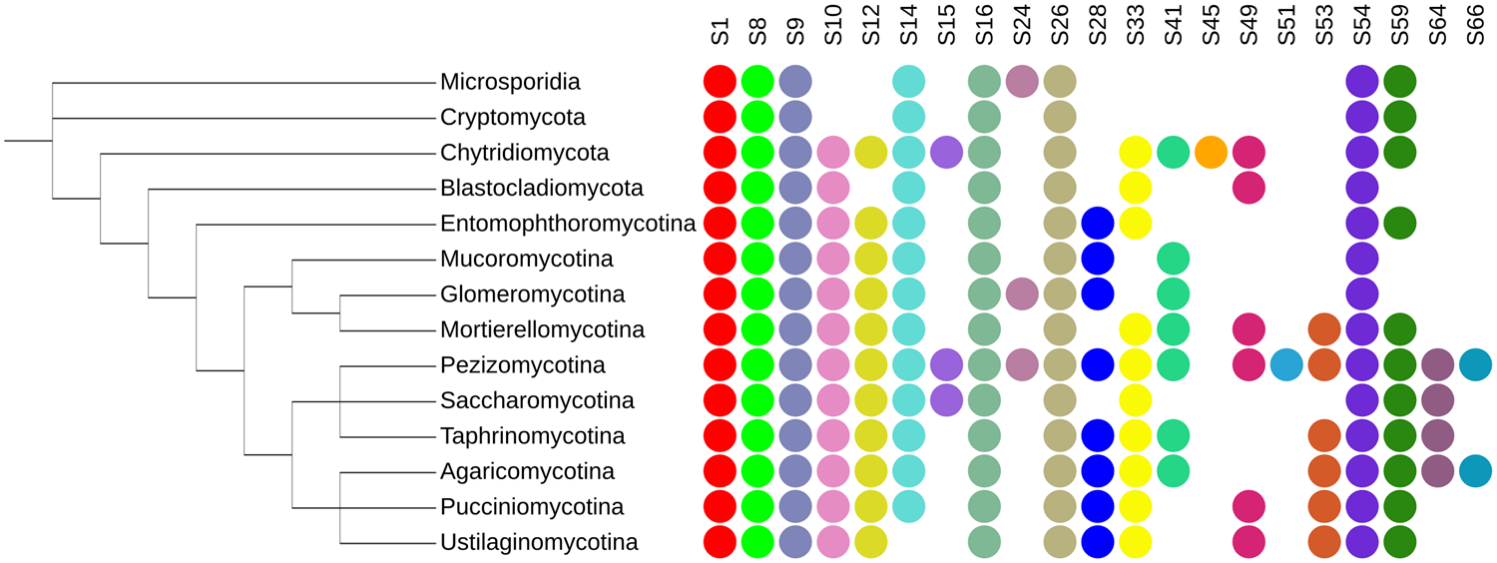
Summary of taxonomic distribution of 21 SP families in Fungi. The image was prepared with iTOL^70^. Schematic tree was drawn based on classification by Spatafora and colleagues^29^.

Recently, we have been witnessing an unprecedented advancement in whole genome sequencing, which embraces also representatives of ancient evolutionary branches and previously understudied taxa, among them many Fungi. In order to identify SPs missing in MEROPS taxonomy for basal fungal lineages, we used a broader NCBI proteome dataset. The resulting SP collection was mapped on the current classification of the Fungal kingdom by Spatafora and colleagues^29^ (see Fig. 2). Our results show that no fungal taxon produces the whole ensemble of 21 SP families. For instance, Agaricomycotina and Pezizomycotina, studied in the greatest detail, lack penicillin G acylase precursor (S45) proteases. The overall limited taxonomic distribution of S45 proteases is likely a result of horizontal gene transfer (HGT) from bacteria to a chytrid *Gonapodya prolifera* JEL478. The latter was the only fungus harbouring two penicillin G acylase precursor (S45) proteases with similarity (35–38% identity with 97–98% coverage) to different proteobacteria and actinobacteria and chloroflexi sequences (see Supplementary Fig. 1a).

Microsporidia, Cryptomycota, Blastocladiomycota and Wallemiales (Agaricomycotina) produce as few as 7–8 types of SPs. Those organisms are often parasites with compact reduced genomes and proteomes. The minimization and specialization of the secretome, which seem to be parallel both in the basal taxa and in the basidiomycete, suggest that S1, S8, S9, S16, S26 and S54 families might be essential for fungi.

### Protease abundance

**Figure 3:**
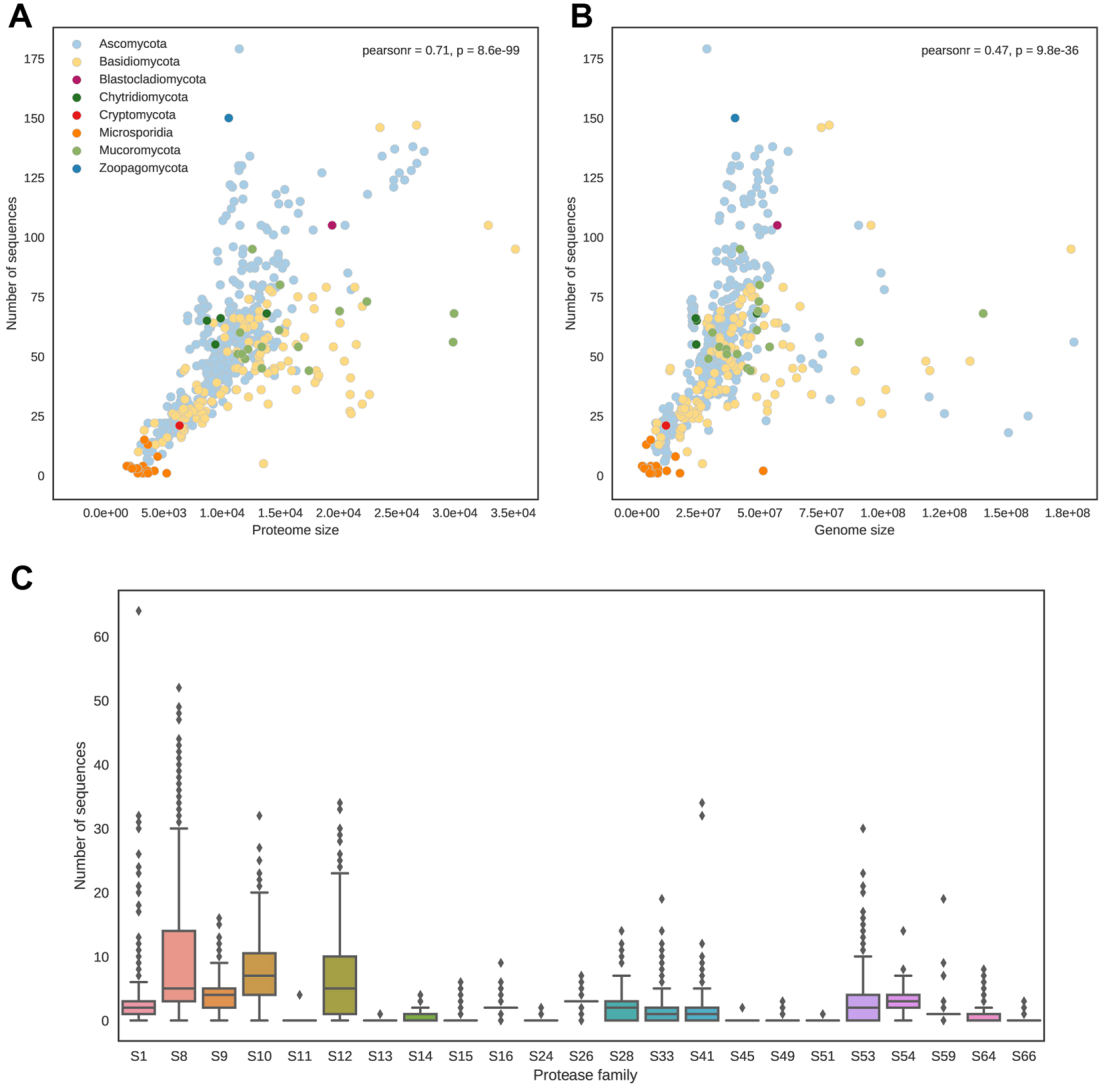
Correlations between SP abundance and proteome (**A**) or genome size (**B**). Points were coloured according to classification to fungal phyla. (**C**) The abundance of SP families within fungal proteomes. The image was prepared in Jupyter Notebook^68^ with matplotlib and seaborn packages.

Proteome size is the main factor impacting the overall abundance of SPs (see Fig. 3A), while genome size correlates to it to a lesser degree (see Fig. 3B). This effect can be explained by the extensive accumulation of non-coding elements in bigger genomes, what accounts for the overall genome size but does not translate into proteome expansion. Serine proteases constitute a considerable fraction of fungal proteomes ranging from 0.02% for *Anncaliia algerae* PRA109 to 1.56% for *Torrubiella hemipterigena*, with a median of 0.43% for all Fungi. Some protease families prevail as single family members per genome, e.g. S14, S15 and S64, whereas others can be found in higher numbers, e.g. the best studied trypsins (S1), subtilisins (S8), sedolisins (S53), D-Ala-D-Ala carboxypeptidase B (S12), carboxypeptidase Y (S10) and C-terminal processing peptidase (S41) (Supplementary Fig. 1b).

### Serine protease repertoire and fungal lifestyle

**Figure 4:**
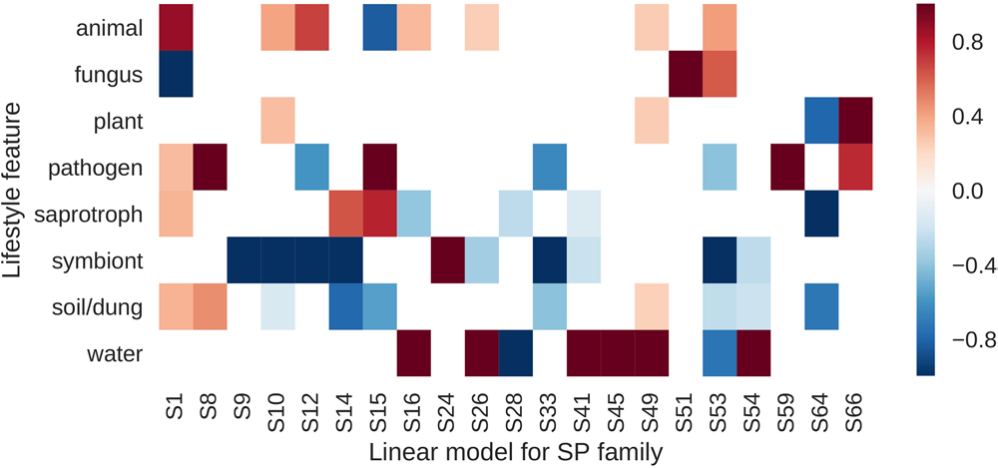
Relationships between defined lifestyle features and serine protease abundance. Colour intensities correspond to the coefficient values of the linear regression models; each column is scaled according to the maximum value; white cells depict no correlation or statistically insignificant relation.

There were single reports on chymotrypsin/trypsin (S1) association with pathogenic lifestyle^28^ and subtilisin (S8) association with saprotrophic lifestyle^25^, however, hypotheses raised there could not be tested with statistical methods due to dataset limitations. In this work, we collected a more comprehensive dataset and applied linear models, decision trees and clustering methods to verify potential lifestyle and protease repertoire associations. We found that general lifestyle resonates with the overall encoded serine protease repertoire (Fig. 4). The most pronounced ecological impact can be noticed for symbiotic fungi and fungi with reduced proteomes – both strategies leading to reduction in SP ensemble (Supplementary File 2 and Supplementary Table 1f).

Fungal lineages with reduced proteomes (Microsporidia, Saccharomycotina, Ustilagomycotina) have few, if any, ClpP endopeptidases (S14), often lack sedolisins (S53) and lysosomal Pro-Xaa carboxypeptidases (S28). Moreover, Ustilagomycotina, Taphrinomycotina, Microsporidia, and Saccharomycotina have scarcely any prolyl aminopeptidases (S33).

Symbiotic fungi have notably less subtilisins (S8), carboxypeptidases Y (S10), D-Ala-D-Ala carboxypeptidases B (S12), LexA (S14), signal peptidase I (S26), sedolisins (S53) and rhomboid (S54) proteases. Most sequenced symbiotic fungi live in association with plant hosts and our understanding of symbiotic adaptation to other hosts is very limited. Interestingly, many of the aforementioned protease families are expanded in fungi, which can be both saprotrophic and pathogenic with the same hosts under specific conditions.

Pathogenic lifestyle has a profound impact on chymotrypsin/trypsin (S1) and subtilisin (S8) expansions, and reduces the number of prolyl aminopeptidases (S33). One of the most striking correlations can be noted for subtilisin (S8) expansion in pathogenic and soil/dung inhabiting fungi regardless of their host preference. Previous studies pointed to an association of high abundance of subtilisins with adaptation to an animal host, but this scenario is not that pronounced for different animal-associated fungi, neither in Eurotiomycetes, nor in Microsporidia. Noteworthy, living in saprotrophic locations such as soil and on dung correlates with an elevated number of subtilisins. This co-occurrence might suggest a necrotrophic application of subtilisins. The same combination of soil/dung habitat and pathogenic lifestyle correlates with expansions of trypsins (S1). Also X-Pro dipeptidyl-peptidase (S15) expanded particularly in pathogenic Hypocreales, Chaetothyriales and Eurotiales. Interestingly this protease is predicted to localize within the cell and has no functional annotation in fungi.

In general, animal-associated fungi tend to have less prolyl oligopeptidases (S9), lysosomal Pro-Xaa carboxypeptidases (S28) and prolyl aminopeptidases (S33).

Plant-associated fungi can be distinguished by their abundant prolyl oligopeptidases (S9), carboxypeptidases Y (S10), D-Ala-D-Ala carboxypeptidases B (S12), prolyl aminopeptidases (S33) and sedolisins (S53). As expected, most of the associations we identified are of a mixed nature of shared ancestry and ecological factors.

### Characteristic features of 21 fungal SP families

Almost all fungal taxa have a core set of proteases comprising S1, S8, S9, S10, S12, S14, S16, S26, S28, S53 and S54, of which S14, S16, S26 and S54 are related to mitochondria while S1, S8, S9, S10, S28 and S53 are often secreted. A particular family can group housekeeping intracellular proteins along with secreted proteases. Most families contain single-domain proteins with only single multidomain architectures. Most of the analysed sequences have well conserved active sites and are likely functional. Table 1 summarizes sequence, function and biological features of all 21 protein families; additional information is provided in Supplementary Table 1 g. Codon usage at the catalytic Ser residue shows an evolutionary preference for TCN codons over AGY codons in families S14, S41, S53, S8, and S66 (see Supplementary Table 1i). The CTG codon characteristic for species of the CTG clade of the subphylum Saccharomycotina is present only in S54 (Rhomboid) family.

Many of the families remain understudied in fungi, particularly those families, which are absent in Saccharomycotina.

### New subfamilies

Clustering of the identified SP sequences revealed the presence of previously unreported subfamilies in four of the families, namely S1, S8, S41 and S54. Their characteristic sequence motives including catalytic residues as well as taxonomic distribution are summarized in Supplementary Table 1h, graphical visualization of sequence clustering is available as Supplementary Figure 1c–f.

#### Trypsin & chymotrypsin (S1)

Trypsin is the archetypal SP present both as a digestive enzyme in animals and as a secreted protease in many microorganisms. From five subfamilies described in MEROPS, 4 (namely A, B, C and E) could be identified in our dataset. Additionally, six more subgroups emerged from clustering (see Supplementary Figure 1c), among them three Pezizomycotina specific subfamilies, one present only in Agaricomycotina, one in Saccharomycotina and a subfamily formed by outgroup sequences from Phytophthora. Supplementary Table 1h summarizes sequence and taxonomy features of the newly identified subfamilies. Trypsin-like proteases were considered as phytopathogenicity markers^28^, yet this might be due to a skewed ratio of saprotroph and pathogen proteomes available for building the dataset. Dubovenko and colleagues also showed the correlation between S1 proteases and pathogenicity against insects and fungi, but not against vertebrates.

#### Subtilisin (S8)

Subtilisins (S8) constitute the most abundant and diverse, in terms of distinguishable subfamilies, family of proteases present in Fungi (Supplementary Figure 1d). Previous studies have identified a number of subfamilies within this huge family, among them kexin (S8B), proteinase K, pyrolysin, osf, tripeptidyl-peptidase II and those labelled new1-new6^30^. Our analysis and recent studies by Li and colleagues^31^ confirmed the presence of new1-4 subfamilies, where new4 should be referred to as tripeptidyl-peptidase II. Subfamilies new5 and new6 were possibly artefacts from mis-predicted protein products. Additionally, we identified a distant subgroup formed by a handful of Pezizomycotina sequences with a unique Asp residue region characterized by a DxExG motif instead of the typical DTG sequence motif. This group maps onto a CDD domain Protease S8 uncharacterized subfamily 11 (cd04843).

#### Programmed cell death in fungi and pathogen adaptation – C-terminal processing peptidase (S41)

S41 proteases constitute a sparsely annotated family with only two subfamilies described in MEROPS: prokaryotic tricorn peptidase (subfamily 41B) and eukaryotic C-terminal processing peptidase (subfamily 41 A). Interestingly, Pfam database includes more than 600 fungal S41 representatives, whereas MEROPS only 4 from 3 taxa: *Cladophialophora immunda*, *Cordyceps bassiana* and *Sebacina vermifera*. A recent article by Iketani and colleagues sheds some light on probable caspase-like function of S41 members based on the Agaricomycotina-conserved protein from *Flammulina velutipes* ^32^.

We identified 972 fungal S41 sequences not only from Agaricomycotina, Taphrinomycotina but also in Ascomycota, Mucormycotina, Mortierellomycotina, Glomeromycotina and *Batrachochytrium dendrobatidis*. If the caspase-like function is conserved across the S41 family, it would point to an ancient origin of the programmed cell death (PCD) in fungi. It has been documented that PCD is required for mushroom development^33^ and unicellular yeasts are used as a model for animal apoptosis^34^. One might think of morphological complexity originating in Mortierellomycotina, but chytrids are simple unicellular organisms and also have an S41 representative, which supports the hypothesis of ancient PCD origin. Interestingly, *B. dendrobatidis* JAM81 and *B. dendrobatidis* JEL423 have 32 and 34 S41 proteases, respectively, whereas the remaining taxa possess 1–2 copies, if any, (except for both *Rhizophagus irregularis* genomes with 4–6 copies). Another hypothesis based on the expression profile of some of the in-paralogs in *B. dendrobatidis* could link members of S41 family to the infection process. In 2008^35^, 29 of the 32 S41 paralogs were identified in the genome, and out of those 12 showed higher levels of expression in sporangia samples versus zoospore samples. The whole set of 32 S41 family members were described as a recent taxon specific duplication in the *B. dendrobatidis* genome^36^. Therefore, S41 proteases might perform very basic roles common to all fungi, and expanded occasionally for specific purposes plausibly connected with infectious capacity. All Batrachochytrium and single *Spizellomyces punctatus* (KNC99789.1) representatives form a separate subfamily based on their sequence divergence compared to canonical S41 proteins and the lack of the otherwise conserved lysine catalytic residue (see Supplementary Figure 1e). Besides, *S. punctatus* retains also canonical S41 proteases.

#### Rhomboid (S54)

Rhomboid (S54) subfamilies correspond to homologs of yeast proteins Pcp1/Rbd1, Rbd2 and *Caenorhabditis elegans* ROM-1 (see Supplementary Figure 1f). Rhomboid evolutionary history is possibly shaped by ancient HGT^37^. These membrane-bound proteases are important for mitochondrial membrane fusion, apoptosis, and stem cell differentiation^38^. Rbd2 is a protein with unknown function found in Ascomycotina and Mucoromycotina, whereas Pcp1/Rbd1 and ROM-1 proteases have a broader taxonomic distribution. The presence of all three groups in Mucoromycotina points to a possible ancient origin and subsequent divergence into three subgroups.

#### A recent fungal innovation Ssy5 (S64)

S64 family seems to be a fungal evolutionary innovation present only in Ascomycota and Agaricomycotina within Basidiomycota. Ssy5 is found solely in Fungi except for 5 sequences classified to Pfam Peptidase_S64 family (PF08192), which includes representatives from Viridiplantae: *Eutrema salsuginueum*, *Physcomitrella patens;* Amebozoa: *Dictyostelium discoideum*; and Metazoa: *Crassostrea gigas*. All five non-fungal proteins display, however, only scarce sequence similarity to the PF08192 family profile (E-value >= 0.01 with HMMer; not detectable with Blast) which questions their S64 membership. Ssy5 peptidases are single domain proteins, predicted to localize into the cytoplasm and nucleus. They belong to Pfam clan Protease_PA and are distantly related to proteases from the chymotrypsin family (S1)^39^. They are also similar to Protease S39 (PF02122; FFAS03 score: −13.8).

In yeast, Ssy5 peptidase processes a transcription factor responsible for signalling that regulates the uptake of extracellular amino acids^40^, and is an element of SPS pathway, which regulates sensing of extracellular amino acids^41^. Ssy5 are limited to Dikarya with most known members identified in Ascomycota and a few members of Agaricomycotina, but not other Basidiomycota (neither Ustilagomycotina nor Pucciniomycotina). The Pfam database harbours only one Agaricomycotina S64 representative from *Pisolithus tinctorius* (NCBI ACC: KIO05838.1), so our results suggest a possible Ssy5 expansion out of Ascomycota. Recently, *Candida albicans* SPS pathway was shown to be important for disabling macrophages^42^ which links food intake to virulence and renders SPS-related enzymes of great interest.

#### A peptidoglycan hydrolase with an atypical history – LD-carboxylpeptidase (S66)

LD-carboxypeptidase Peptidase_S66 (PF02016) is a predominantly bacterial family of proteases capable to hydrolyze the peptide bond between L- and D-amino acids in bacterial peptidoglycan^43^. They are thought to be involved in peptidoglycan recycling^44^. Surprisingly, some LD-carboxypeptidases are also present in a handful of Dikarya and in other Eukaryotic kingdoms: Metazoa (*Acyrthosiphon pisum* UNIPROT: J9K870, × 1WVY8, × 1 × 7C1), Amebozoa (*Acanthamoeba castellanii* UNIPROT: L8H6R2), Oomycota (11 taxa) and Viridiplantae (*Physcomitrella patens* UNIPROT: A9T2B7, A9U581). The ability to degrade bacterial cell wall components might be important for the interaction with pathogenic and symbiotic bacteria. The observed patchy taxonomic distribution might be a result of either multiple deletions or multiple horizontal gene transfers. An independent HGT from bacteria to aphids and fungi (*Gibberella zeae* sequence, RefSeq: XP_383840) has been proposed by Nikoh and Nakabachi^41, 45^. A phylogenetic tree inferred for S66 sequences is not congruent with fungal taxonomy. Bacterial sequences are grouped with sequences from other domains of life, and seven Archaea sequences are split into 3 distant clades. Most of fungal sequences are grouped in a well separated clade sister to an Oomycota clade and a single extremophilic archeon *Natrinema* sp. sequence (UNIPROT:I7CEN7). Four other fungal sequences from *Capronia epimyces* (EXJ84096.1), *Serendipita vermifera* (KIM20056.1, KIM25268.1) and *S. indica* (CCA67026.1) form a well defined clade in an unresolved part of the tree (see Supplementary Figure 1g). Moss (*Physcomitrella patens*) sequences are present in two distant clades and aphid (*Acyrthosiphon pisum*) sequences group together with invertebrate endosymbiont sequences (*Orientia tsutsugamushi*, *Hamiltonella defensa*). A single compact fungal clade suggests a single transfer from unknown bacteria to a filamentous Acomycete. The presence of Archaea, Oomycota and Plantae in distant parts of the tree may be a hallmark of multiple HGT events.

## Discussion

The aim of this project was to explore serine protease abundance across and in the context of the fungal kingdom taxonomy. This is a bold objective, given the unresolved evolutionary relationships between the major fungal taxa. Both higher and lower branches in the fungal tree of life, are still under construction. Especially, the history of early diverged fungal organisms requires further elucidation. The earliest branches of Fungi are in order of divergence: Cryptomycota and Microsporidia with an obligate parasitic lifestyle, Chytrydiomycota with posteriorly-uniflagellate zoospores and Blastocladiomycota with hyphae that is better developed that the one in Chytrydiomycota^46^. The first to lose the flagellum and become terrestrial were organisms belonging to: Mucoromycota and Zoopagomycota^29, 47^. Ascomycota and Basidiomycota constituting the vast majority of all described fungi are considered to be evolutionarily the youngest.

In order to determine what is the protease composition of fungal proteomes, we carried a global search of serine proteases in publicly available proteomes. All Fungi, being osmotrophs, extracellularly degrade organic matter to obtain nutrients and the secreted enzymes are crucial for their survival. Therefore, one might anticipate an unprecedented diversity of SP families here. However, our results show that most of serine protease repertoire present in fungal proteomes is also present in other eukaryotic lineages, pointing to a common origin of serine protease families, especially considering those related to mitochondria, which likely derive from the last common ancestor of eukaryotes and are shared with bacteria. Fungi possess only one evolutionary SP innovation: the Ssy5 protease (S64) involved in amino acid sensing^40^, which most likely originated in Dikarya or was lost in lineages that diverged earlier. All remaining SP families are shared with other evolutionary lineages of eukaryotes but not always with the closest relatives within Opisthokonta, e.g. many animals and *Fonticula alba* lack sedolisins. The SP distribution is not uniform across Fungi. While some proteases are present ubiquitously, e.g. housekeeping enzymes, other proteases, especially the secreted ones, are related to certain trophic strategies. The overall abundance of proteases is strongly correlated with proteome size and somewhat correlated with genome size. Especially plant-related fungi show elevated numbers of proteases, whereas animal-related fungi are less prone to protease expansions. There are, however, exceptions from this rule, one of which is a huge expansion of C-terminal processing peptidases in *B. dendrobatidis* but absent in other sequenced chytrids. Symbiotic fungi, on the other hand, represented mostly by plant symbionts in our dataset, have lower numbers of secreted proteases, consistently with the previously published results on mycorrhizal fungi, which reduces the potential of degrading their host^48^ in order to decrease the defence reaction on the plant side and to benefit from more accessible nutrient source mutually established in the course of co-evolution. A different strategy is present in pathogens and saprophytes, which are often good degraders and possess host-adapted toolkits to gain access to nutrients. Among them, pathogenic fungi living in the soil and on dung have more subtilisins and trypsins than other groups, what is partially in agreement with previously formulated hypotheses^28^. However, we did not find a statistical signal pointing at a preference for subtilisin and trypsin overrepresentation with a host type, neither plant nor animal. Other proteases e.g. prolyl oligopeptidases (S9) were less abundant in animal-associated fungi and more numerous in plants. Prolyl oligopeptidases constitute a diverse and ubiquitous group of enzymes, which comprises among others cytosolic prolyl oligopeptidase and membrane-bound dipeptidyl peptidase IV^49^, one of the most abundant SPs in fungal genomes.

The lack of support for host preference in subtilisin and trypsin abundance datasets might be a consequence of predominant taxonomy effect. Multiple lifestyles, as diverse as dermatophytes, entomopathogens, opportunistic animal pathogens and nematode predatory fungi can be represented in one taxon e.g. Pezizomycotina formed by species of similar proteome size, the latter being the strongest factor affecting SP abundance. Moreover, the broad category of animal host defined in this work additionally masks the natural diversity of animal hosts (vertebrates, insects, nematodes, acarids). The effect of proteome size in its extreme can be observed for organisms with reduced proteomes, among them Microsporidia, which live as obligate intracellular parasites, Saccharomycotina adapted to sugar-rich environments and Ustilaginomycotina, which often live as obligate biotrophs, encode only a minimal ensemble of proteases with low copy numbers.

Our findings point to several previously unreported correlations between serine protease abundance and fungal life strategies, which provide basis for further studies.

Current data aggregation and analysis are hindered by our limited understanding of fungal ecology and complex relationships between fungi and their endosymbiotic bacteria and eukaryotic hosts. We are just scratching the surface of multispecies interactions rarely taking spatiotemporal changes into account. A single fungus is capable of both endophytic plant colonization and successful insect invasion by using its versatile enzymatic toolkit. Defining this toolkit is a very basic but critical step towards thorough understanding of fungal ecology, which cannot be achieved without further studies involving molecular and environmental biology.

## Methods

### Dataset and sequence searches

MEROPS^4^ libraries were downloaded in October 2016 and mapped on Pfam 30 database of protein domains using Pfam_scan.pl^50^ as a wrapper for hmmscan^51^. This resulted in a unequivocal MEROPS to Pfam relation for 40 out of 52 SP families (52 instead of 54, because S63 was moved to P2 family and S67 entry is empty). Some MEROPS protease families include more than one Pfam family eg. chymotrypsin (S1) proteases belong to Trypsin (PF00089) and Trypsin_2 (PF133645) families. On the other hand, certain protein families are narrower in MEROPS than in Pfam, for example subtilisin (S8) and sedolisin (S53) proteases belong to one Pfam protein family, Peptidase_S8 (PF00082). The mapping between Pfam and MEROPS families is available as Supplementary Table 1c. Three MEROPS protease families: S67, S79 and S80 could not be assigned to Pfam and their sequence profiles were built using corresponding MEROPS sequences. Family S69 has only one representative given in MEROPS and we couldn’t identify any further homologs.

Fungal proteomes were downloaded from NCBI on 17^th^ August 2016^52^ and were screened with hmmscan against 51 sequence profiles (S69 had no homologs). Additionally, full proteomes of 20 representatives from other eukaryotic kingdoms were downloaded from NCBI and processed in parallel with fungal proteomes in order to provide a reference and outgroup for further analyses (a list of analysed proteomes is presented in Supplementary Table 1a). The taxonomic distribution of MEROPS families was copied directly from the MEROPS website (Supplementary Table 1b). However, MEROPS counts in all homologs, including those truncated, inactive or even with a different enzymatic activity and lacking protease activity (see family S33), which blurs the true distribution of proteases.

### Clustering and dataset curation

In order to elucidate the relationships between and within SP families, graphical clustering was performed with CLANS. CLANS^53^ is a Java-based clustering tool which visualizes pairwise sequence similarities and enables iterative comparisons of sequence datasets. The graphical representation depicts proteins as vertices and pairwise Blast mappings as edges. Such a representation enables outlier identification and eases the dataset curation.

Each of the fungal protein families was clustered together with: i) Pfam seed representative sequences, ii) MEROPS representative sequences, iii) eukaryotic model organisms sequences, and iv) PDB representative sequences. Sequences belonging to one Pfam clan were clustered together in order to ensure proper classification and observe relationships between subfamilies and families.

Outlier sequences were carefully inspected to remove false positive hits. Dubious hits were analyzed using SMART^54^, CD-search NCBI^52^, InterProScan^55^ and HHpred^56^, and removed if results revealed non-specific mappings.

### Sequence analysis and phylogeny inference

Sequences belonging to each protein family were aligned using Mafft iterative alignment method^57^. Each alignment was manually curated, all proteins with deletions in the conserved regions of the enzymatic domain and/or with mutations replacing 2 of 3 catalytic residues were excluded. The curation process was assisted by sequence to structure mappings using Meta-BASIC^58^ and FFAS03^59^. PDB references were used, where available.

Each family was additionally clustered separately in CLANS with respective Pfam seed, MEROPS representatives, PDB representatives and model Eukaryote outgroup sequences. This second round of clustering was carried out for a graphical inspection of relationships between subfamilies and sequence distribution of particular taxonomic groups.

Phylogenetic trees were inferred only for the protease families where horizontal gene transfer might be responsible for atypical taxonomic distribution. Sequence alignments were prepared in Mafft (linsi, 100 iterations)^57^, dubious regions were removed by TrimAl trimming^60^ (automated1). The best model for phylogenetic analysis was chosen based on AIC and BIC criteria in ProtTest^61^. Maximum likelihood phylogenetic inference was carried in PhyML^62^.

Domain architecture (Pfam_scan.pl^50^), protein localization ((WoLF PSORT^63^, TargetP^64^) and transmembrane regions (TMHMM^65^) were assessed for all members of all identified fungal families of SPs. Codon usage analysis was performed based on GFF files, significance of observed differences was evaluated with Fisher’s exact test.

### Statistical analysis

The dataset for fungal lifestyle description was built based on the available literature. Each fungus was assigned to separate categories, including host type (plant, animal, fungus) main habitat (soil/dung, water) and lifestyle (pathogenic, symbiotic and saprotrophic). One organism could be assigned to multiple categories if was able to live for example both as a plant symbiont and animal pathogen. The taxonomy assignment was based on NCBI taxonomy database with manual fine tuning.

Linear models incorporating taxonomic and lifestyle features for fungal genomes were built in Python with the statsmodels package^66^. Taxonomic features were constructed at class level for Agaricomycotina and Pezizomycotina and at subphylum level for smaller taxa to obtain groups of a more comparable size. Exploratory analysis and basic statistics for the dataset were carried out using pandas and seaborn. One-hot encoding was used to represent categories, and categories with less than 5 observations were not included. In order to retain maximum biological information for taxa with less than 5 members, lifestyle features were kept, while taxonomic categories were replaced with a null vector. Proteome and genome sizes were scaled by their maximum value. Linear models were built on taxonomic and lifestyle data, proteome and genome sizes and protease count as predicted variables. Stepwise regression was used and insignificant features were eliminated based on t-test (p-value threshold equal 0.05).

Decision trees predicting proteases count were built on taxonomic and lifestyle data with scikit-learn^67^ package using default parameters, with up to 5 levels of depth and at least 5 samples in each leaf. Mean squared error was used as the impurity measure and a threshold of 0.01 was used for splits.

The whole dataset is available as Supplementary Table 1e and code for statistical procedures is available as a Jupyter Notebook^68^ Supplementary File 2.

## Electronic supplementary material

Supplementary Figures and Legends

Supplementary File 1 and Supplementary File 2

Supplementary Table 1
