# Supplementary material for: Fungal lifestyle reflected in serine protease repertoire
Source: Sci Rep. 2017 Aug 22;7:9147. doi: 10.1038/s41598-017-09644-w (PMC5567314; doi:10.1038/s41598-017-09644-w)
Supplement: Supplementary file 1 — Supplementary Figures and Legends [file 41598_2017_9644_MOESM1_ESM.pdf]

Supplementary Data

Fungal lifestyle reflected in serine protease repertoire

Anna Muszewska[1,*], Marta M. Stepniewska-Dziubinska[1], Kamil Steczkiewicz[2], Julia Pawlowska[3], Agata Dziedzic[1], Krzysztof Ginalski[2]

[1] Institute of Biochemistry and Biophysics, Polish Academy of Sciences, Pawinskiego 5A, 02-106 Warsaw, Poland

[2] Laboratory of Bioinformatics and Systems Biology, CeNT, University of Warsaw, Zwirki i Wigury 93, 02-089, Poland

[3] Department of Molecular Phylogenetics and Evolution, Faculty of Biology, Biological and Chemical Research Centre, University of Warsaw, Zwirki i Wigury 101, 02-089 Warsaw, Poland

Corresponding author:
*musze@ibb.waw.pl

Table of Contents

Supplementary File 1 3
Supplementary File 2 3
Supplementary Table 1 3
Supplementary Figure 1a 4
Supplementary Figure 1b 5
Supplementary Figure 1c 6
Supplementary Figure 1d 7
Supplementary Figure 1e 8
Supplementary Figure 1f 9
Supplementary Figure 1g 10

Supplementary File 1

Phylogenetic trees for S45 and S66 SP families in the Newick format and the lists of all protein accession numbers for each family of SPs identified in this study.

Supplementary File 2

Jupyter Notebook in html with code, images and statistics used for all statistical analyses discussed in this work.

Supplementary Table 1

In consecutive spreadsheets, the table provides: **a)** the list of proteomes downloaded from NCBI genomes in August 2016 with corresponding literature references, **b)** taxonomic distribution of SP families in MEROPS in October 2016, **c)** Pfam to MEROPS family mapping, **d)** detailed protease counts per genome together with lifestyle and taxonomic description for each organism, **e)** distribution and abundance of SP in 20 model Eukaryota, **f)** characteristic features of newly identified subfamilies, **g)** linear model coefficients and p-values for each protease and variable, **h)** an extended summary of characteristic features of 23 SP families identified in fungi including prediction of domain architecture, subcellular localization, transmembrane elements and secretion signal, **i)** serine codon usage in SP families across fungal subphyla and calculated statistical significance of observed differences between the catalytic serine residue codon and all serine codons evaluated with Fisher's exact test.

Supplementary Figure 1a

Phylogenetic tree of two *Gonapodya prolifera* sequences with the closest bacterial representatives, a hypothetical case of HGT. The tree was built with PhyML, substitution model chosen with Prottest (LG+G+I), aLTR branch supports, image prepared with iTOL. Tree was rooted with Proteobacteria clade.

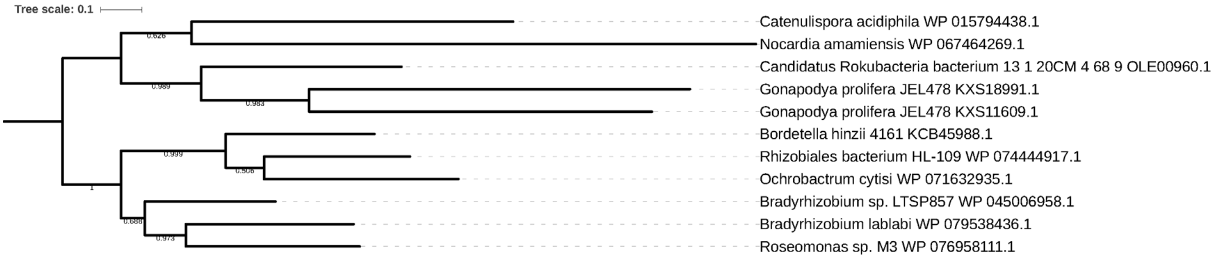

Supplementary Figure 1b

Distribution of SP abundance across fungal species. The figure was prepared using Jupyter.

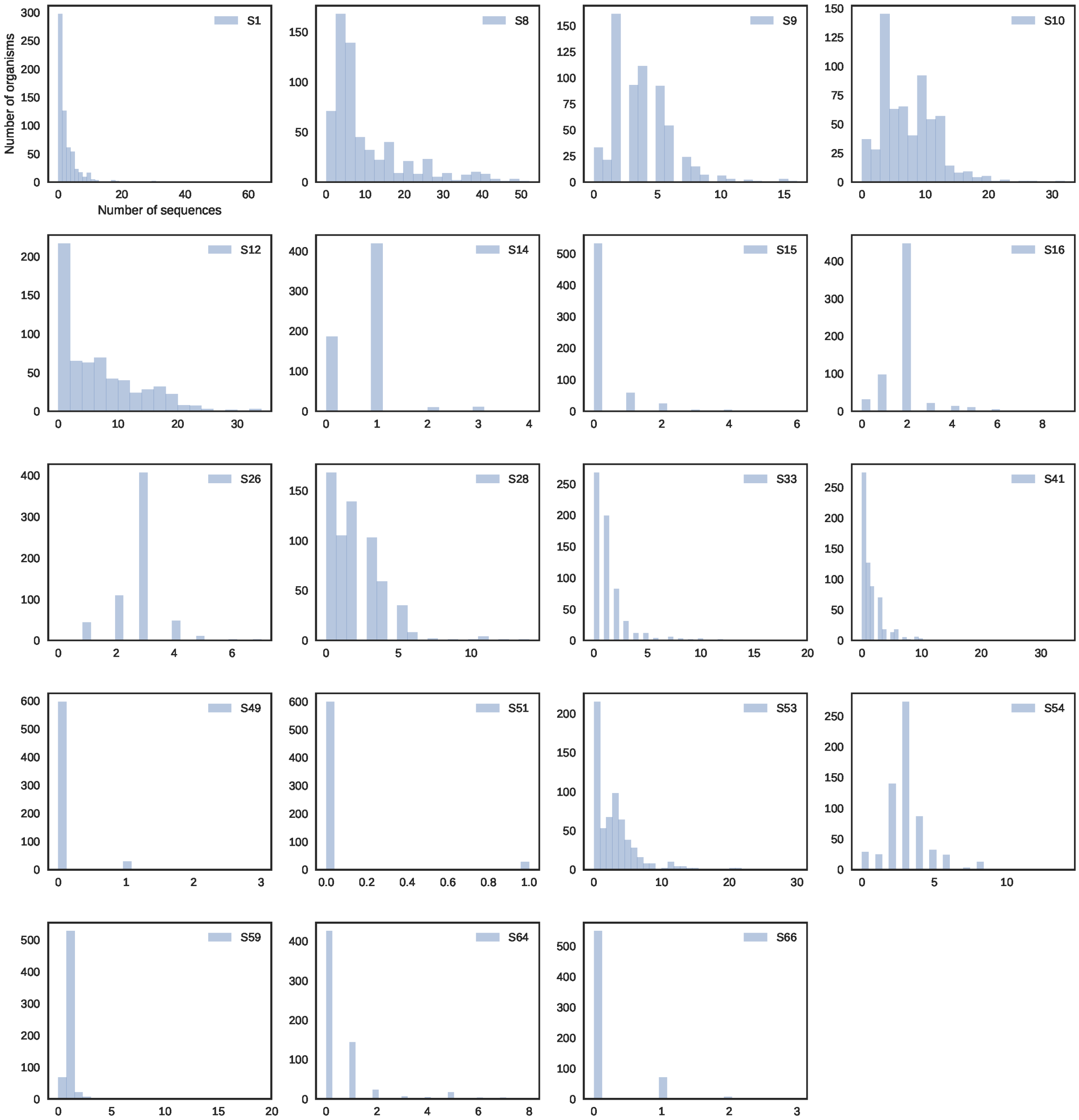

Supplementary Figure 1c

CLANS clustering showing all identified subfamilies in Trypsin/Chymotrypsin (S1) family. Fungal and outgroup sequences are colored according to taxonomical classification. PDB and MEROPS representatives, and Pfam reference sequences are denoted with separate colors. Main subfamilies are labeled.

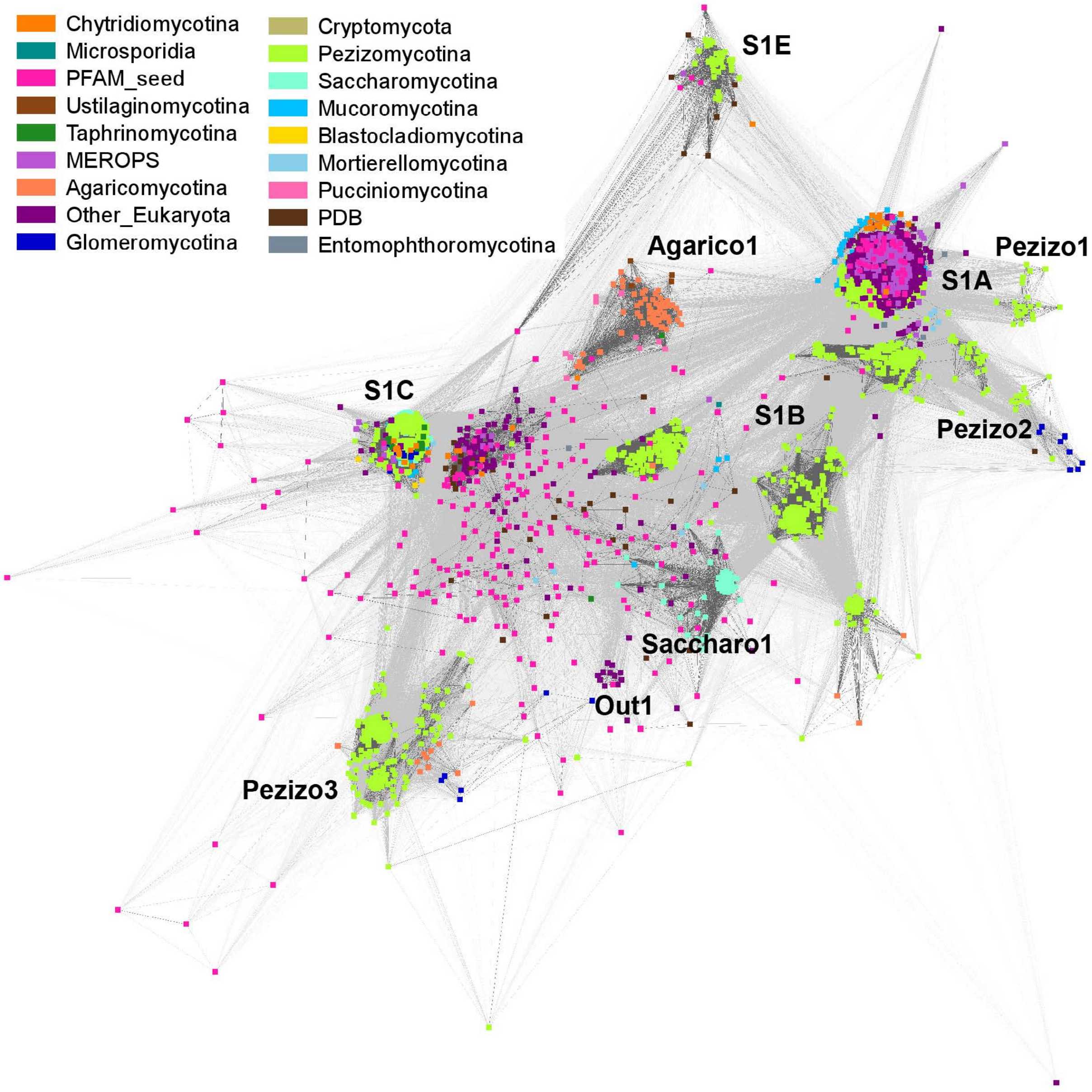

Supplementary Figure 1d

CLANS clustering showing all identified subfamilies in Subtilisin (S8) family. Fungal and outgroup sequences are colored according to taxonomical classification. PDB and MEROPS representatives, and Pfam reference sequences are denoted with separate colors. Main subfamilies are labeled.

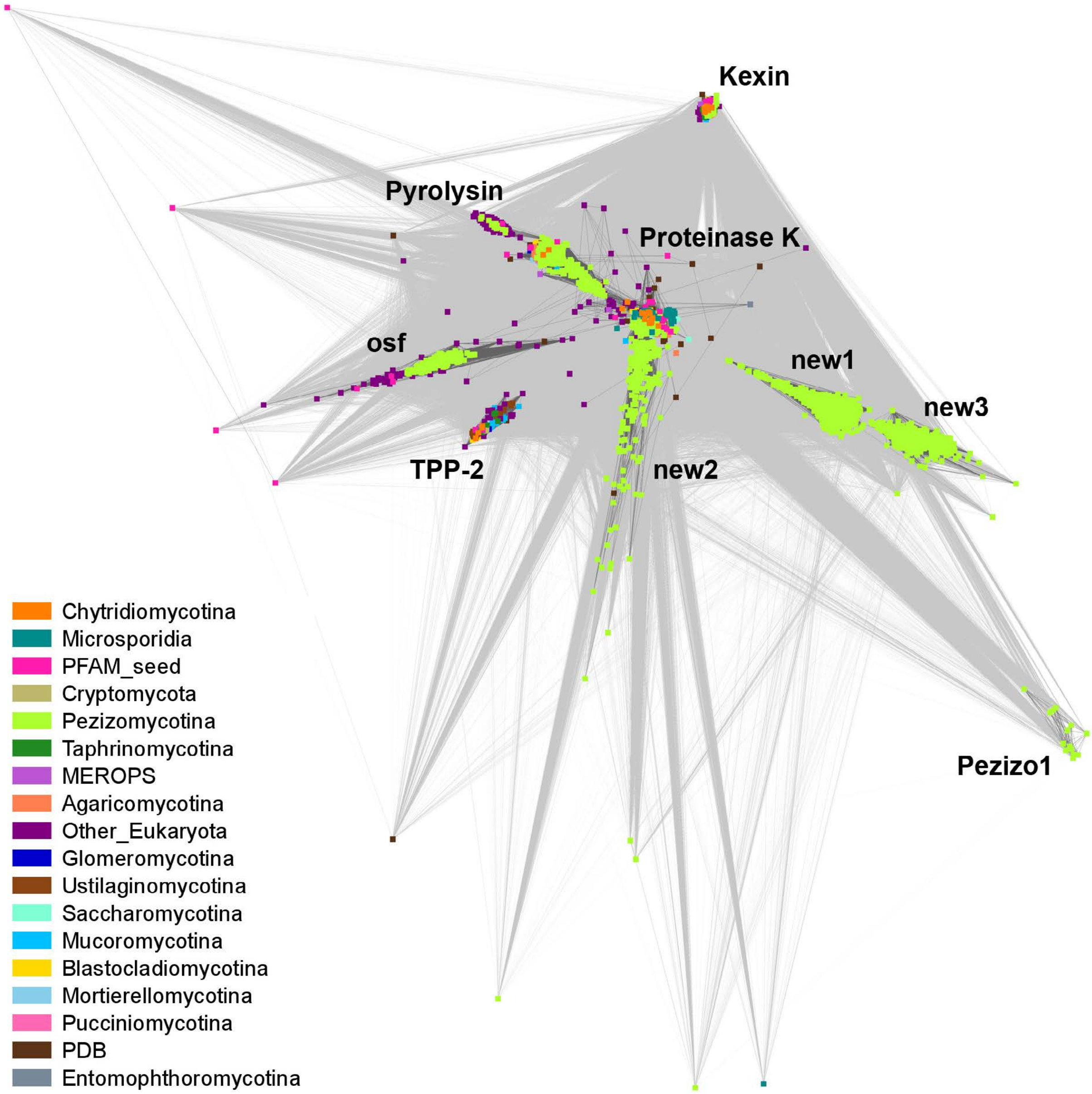

Supplementary Figure 1e

CLANS clustering showing all identified subfamilies in C-terminal processing peptidase (S41) family. Fungal and outgroup sequences are colored according to taxonomical classification. PDB and MEROPS representatives, and Pfam reference sequences are denoted with separate colors. Main subfamilies are labeled.

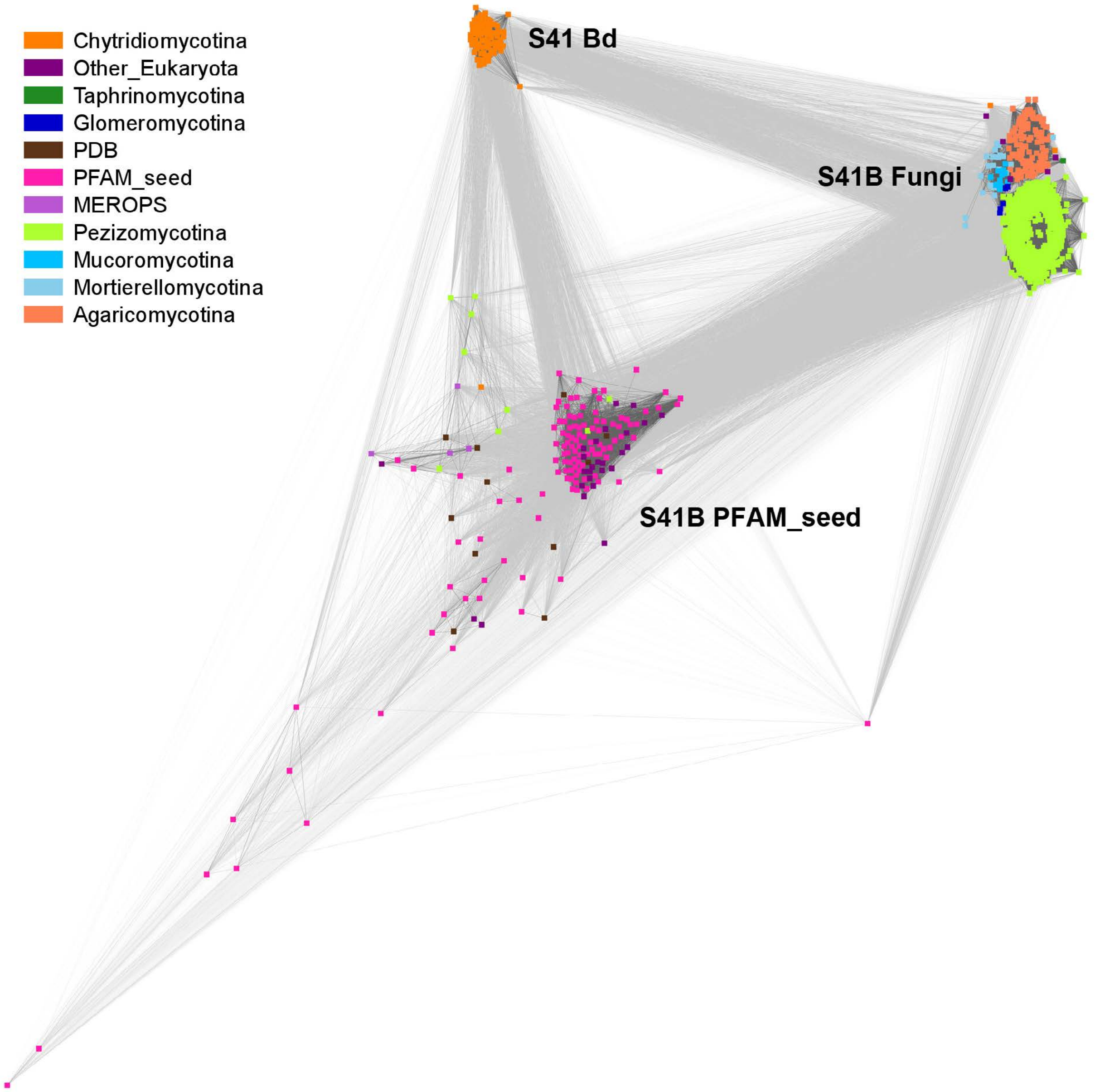

Supplementary Figure 1f

CLANS clustering showing all identified subfamilies in Rhomboid (S54) family. Fungal and outgroup sequences are colored according to taxonomical classification. PDB and MEROPS representatives, and Pfam reference sequences are denoted with separate colors. Main subfamilies are labeled.

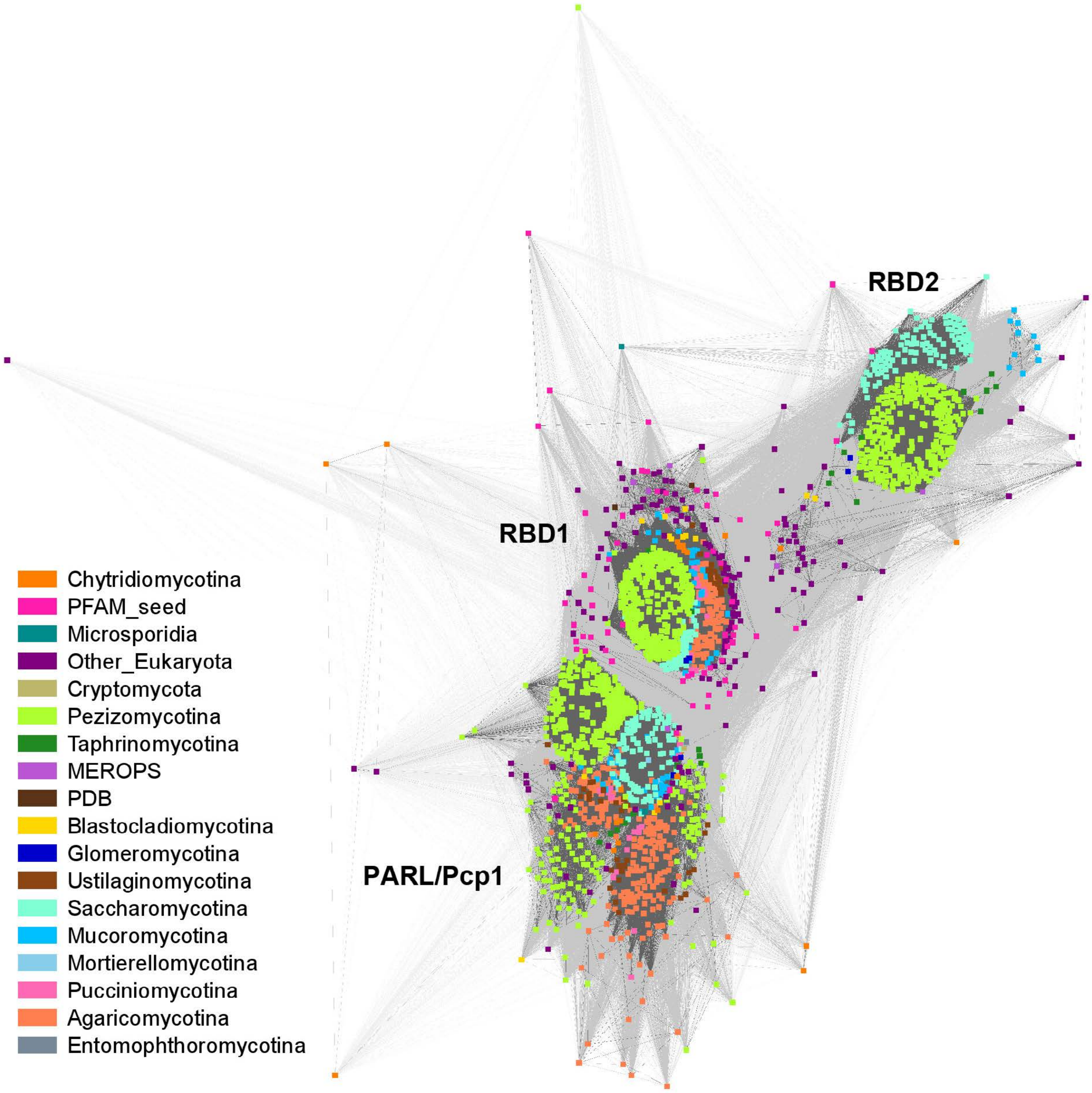

Supplementary Figure 1g

Maximum likelihood phylogenetic tree of 355 sequences inferred using PhyML, LG+I+G model was chosen with Prottest branches with aLTR supports below 0.5 collapsed. Plant sequences are marked in green, fungal sequences in dark red, animal in purple, oomycota in dark blue. Eukaryotic sequences are all in bold, Archaea dark brown and italic, bacterial symbionts of insects are marked in light purple. The image was prepared with iTOL.

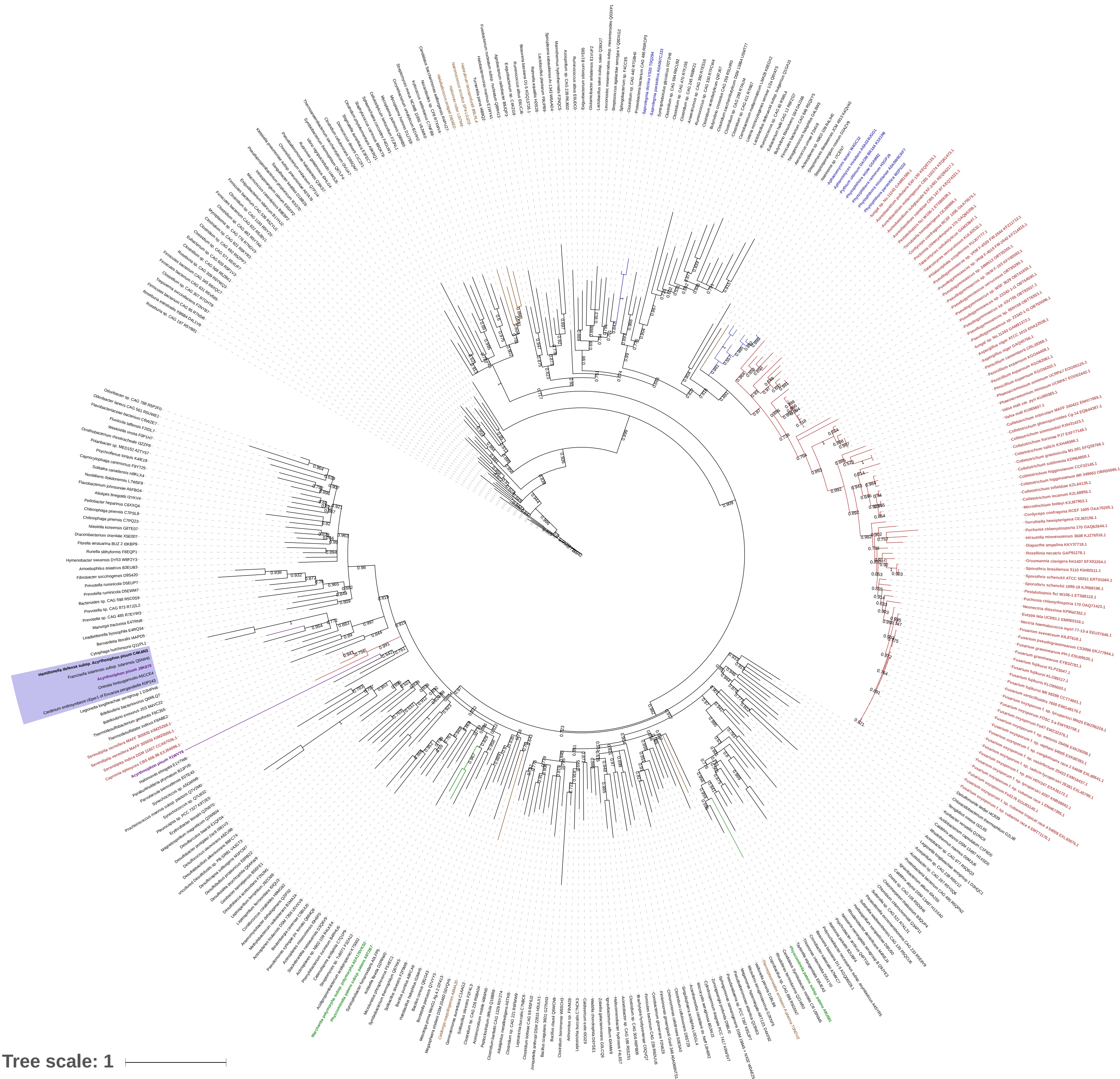
Tree scale: 1
