# Supplementary material for: Fungal lifestyle reflected in serine protease repertoire
Source: Sci Rep. 2017 Aug 22;7:9147. doi: 10.1038/s41598-017-09644-w (PMC5567314; doi:10.1038/s41598-017-09644-w)
Supplement: Supplementary file 2 — Supplementary File 1 and Supplementary File 2 [file 41598_2017_9644_MOESM2_ESM.zip › Supplementary_Files_1_and_2/Supplementary File 2.html]

supplementary\_file


In [1]:

```
import math
import numpy as np
import scipy
import pandas as pd
pd.options.display.max_columns = 25
pd.options.display.max_colwidth = 150

from sklearn import metrics, tree
import statsmodels.api as sm

import pydotplus

from IPython.display import display, Image, SVG

import matplotlib.pyplot as plt
import seaborn as sns
sns.set_style('white')
sns.set_color_codes()


%matplotlib inline
```

# Data preparation¶

Read the dataset, define groups of related column, set dtypes and clean the table.

In [2]:

```
data = pd.read_table('dataset.tab', dtype='object')
```

In [3]:

```
# Groups of related columns
proteases = [col_name for col_name in data.columns if col_name[0] == 'S']

taxonomy = ['phylum', 'subphylum', 'class', 'order', 'family', 'genus', 'organism_name',
            'species taxid', 'strain taxid']

host = ['animal', 'fungus', 'plant']
interaction_type = ['pathogen', 'saprotroph', 'symbiont']
environment = ['soil/dung', 'water']
lifestyle = host + interaction_type + environment

genome = ['genome_size', 'proteome_size', 'assembly_ID']
```

In [4]:

```
# Convert numerical and binary variables to float32
numeric = proteases + lifestyle + ['sum', 'genome_size', 'proteome_size']
data[numeric] = data[numeric].astype(np.float32)
```

In [5]:

```
# Remove records without proteases or without taxonomy
data = data.loc[(data['sum'] != 0) & (~data['genus'].isnull())]
data = data.reset_index(drop=True)

# Save cleaned dataset
data.to_csv('dataset_cleaned.tab', index=False, sep='\t')
```

# Exploratory analysis¶

In [6]:

```
# Plot number of proteases for each record
fig, ax = plt.subplots(figsize=(12, 6))
sns.heatmap(data[proteases].T, xticklabels=False, cmap=plt.cm.bone_r);
ax.set_ylabel('Protease family')
ax.set_xlabel('Organism');
```

In [7]:

```
# Get number of organism coding at least one protease from a given family
num_organisms = (data[proteases] > 0).sum()
num_organisms
```

Out[7]:

```
S1     519
S8     621
S9     594
S10    596
S11      1
S12    477
S13      1
S14    441
S15     95
S16    596
S24      3
S26    625
S28    459
S33    359
S41    353
S45      1
S49     31
S51     28
S53    412
S54    598
S59    559
S64    201
S66     78
dtype: int64
```

In [8]:

```
# ...and as a percent of all records
(100 * num_organisms / len(data)).round(1).astype(str) + ' %'
```

Out[8]:

```
S1     82.8 %
S8     99.0 %
S9     94.7 %
S10    95.1 %
S11     0.2 %
S12    76.1 %
S13     0.2 %
S14    70.3 %
S15    15.2 %
S16    95.1 %
S24     0.5 %
S26    99.7 %
S28    73.2 %
S33    57.3 %
S41    56.3 %
S45     0.2 %
S49     4.9 %
S51     4.5 %
S53    65.7 %
S54    95.4 %
S59    89.2 %
S64    32.1 %
S66    12.4 %
dtype: object
```

In [9]:

```
# Plot distribution of number of proteases

fig, ax = plt.subplots(figsize=(12, 6))
sns.boxplot(data=data[proteases])
ax.set_xlabel('Protease family')
ax.set_ylabel('Number of sequences')

fig.savefig('prot_num_boxplot.pdf');
```

In [10]:

```
# Plothistograms for proteases coded by at least 10 organisms

proteases_to_plot = num_organisms[num_organisms > 10].index

cols = 4
rows = math.ceil(len(proteases_to_plot) / cols)

fig, axs = plt.subplots(ncols=cols, nrows=rows, figsize=(16, 3.5*rows))
plt.subplots_adjust(hspace = .3)
axs = axs.flatten()

for col_name, ax in zip(proteases_to_plot, axs):
    sns.distplot(data[col_name], ax=ax, kde=False, norm_hist=False)
    ax.set_xlabel('')
    ax.legend([col_name])

empty_axes = len(axs) - len(proteases_to_plot)

axs[0].set_xlabel('Number of sequences')
axs[0].set_ylabel('Number of organisms')

for ax in axs[-empty_axes:]:
    ax.set_visible(False)

fig.savefig('prot_num_hist.pdf')
```

In [11]:

```
# Cluster organisms by lifestyle

g = sns.clustermap(data[lifestyle].dropna(), cmap=plt.cm.YlGnBu, yticklabels=False, figsize=(7, 7))
g.ax_heatmap.set_ylabel('Organisms');
g.ax_heatmap.set_xlabel('Lifestyle');
```

```
/home/martas/miniconda/envs/deep_learning/lib/python3.5/site-packages/matplotlib/cbook.py:136: MatplotlibDeprecationWarning: The axisbg attribute was deprecated in version 2.0. Use facecolor instead.
  warnings.warn(message, mplDeprecation, stacklevel=1)
```

# Number of proteases vs genome complexity¶

Plot number of proteases as a function of proteome and genome sizes and color organisms by phylum.
Compute Pearson's correlation coefficient.

In [12]:

```
phylum_colors = {
    'Ascomycota': (0.65, 0.80, 0.9),
    'Basidiomycota': (1.00, 0.85, 0.50),
    'Blastocladiomycota': (0.70, 0.10, 0.40),
    'Chytridiomycota': (0.15, 0.45, 0.15),
    'Cryptomycota': (0.90, 0.100, 0.10),
    'Microsporidia': (1.0, 0.50, 0.0),
    'Mucoromycota': (0.55, 0.70, 0.40),
    'Zoopagomycota': (0.15, 0.50, 0.70)
}
```

In [13]:

```
coeff, pval = scipy.stats.pearsonr(data['proteome_size'], data['sum'])

fig, ax = plt.subplots(figsize=(7, 7))

for phylum, subtable in data.groupby('phylum'):
    ax.scatter(subtable['proteome_size'], subtable['sum'], color=phylum_colors[phylum], edgecolors=(.7, .7, .7),
               label=phylum)

ax.set_xlabel('Proteome size')
ax.set_ylabel('Number of sequences')
ax.text(0.8, 0.95, 'pearsonr = %.2f, p = %.1e' % (coeff, pval), ha='center', va='center', transform=ax.transAxes)
ax.set_ylim(-10,)
ax.set_xlim(-4.5e3,)
ax.set_xticklabels(('%2.1e' % l for l in ax.get_xticks()))
ax.legend(loc='upper left')
fig.savefig('proteases_vs_proteome.pdf');
```

In [14]:

```
coeff, pval = scipy.stats.pearsonr(data['genome_size'], data['sum'])

fig, ax = plt.subplots(figsize=(7, 7))

for phylum, subtable in data.groupby('phylum'):
    ax.scatter(subtable['genome_size'], subtable['sum'], color=phylum_colors[phylum], edgecolors=(.7, .7, .7),
               label=phylum)

ax.set_xlabel('Genome size')
ax.set_ylabel('Number of sequences')
ax.text(0.8, 0.95, 'pearsonr = %.2f, p = %.1e' % (coeff, pval), ha='center', va='center', transform=ax.transAxes)
ax.set_ylim(-10,)
ax.set_xlim(-1e7,)
ax.set_xticklabels(('%2.1e' % l for l in ax.get_xticks()))
fig.savefig('proteases_vs_genome.pdf');
```

# Relationship between lifestyle, taxonomy and number of proteases¶

Encode taxonomy as a set of binary variables (one-hot encoding).
For Pezizomycotina and Agaricomycotina use class, for under-represented taxa use subphylum.

In [15]:

```
# Get list of subphyla and remove Pezizomycotina and Agaricomycotina - those groups are too big and diverse,
# so we will use class instead
subphylum_keys = list(data['subphylum'].unique())
subphylum_keys.remove('Pezizomycotina')
subphylum_keys.remove('Agaricomycotina')

# Use one-hot encoding - each subphylum is represented as a separate binary vector
subphylum_onehot = (data['subphylum'].values[:, None] == subphylum_keys)

# For Pezizomycotina and Agaricomycotina use class to describe taxonomy
class_keys = list(data[data['subphylum'] == 'Pezizomycotina']['class'].unique()) \
             + list(data[data['subphylum'] == 'Agaricomycotina']['class'].unique())

class_onehot = (data['class'].values[:, None] == class_keys)

# Combine encoded taxonomy with other features
encoded = pd.concat((data, pd.DataFrame(subphylum_onehot, columns=subphylum_keys),
                     pd.DataFrame(class_onehot, columns=class_keys)), axis=1)

# Remove redundant columns
del encoded['subphylum']
del encoded['class']

# Remove rows with missing values
encoded = encoded.dropna()

# Get number of organisms in each taxonomic group
group_size = encoded[subphylum_keys+class_keys].sum()

# Do not use taxonomic groups with less than 5 representatives
tax_groups = list(group_size[group_size >= 5].index)

features = lifestyle + tax_groups
print('features:', features)
X = encoded[features].astype(float)
```

```
features: ['animal', 'fungus', 'plant', 'pathogen', 'saprotroph', 'symbiont', 'soil/dung', 'water', 'Saccharomycotina', 'Taphrinomycotina', 'Pucciniomycotina', 'Ustilaginomycotina', 'Microsporidia', 'Mucoromycotina', 'Dothideomycetes', 'Eurotiomycetes', 'Leotiomycetes', 'Sordariomycetes', 'Agaricomycetes', 'Tremellomycetes']
```

Compute conditional relationships between binary features, where different rows correspond to different conditions.

We can see that e.g. all Microsporidia are pathogens (dark color for row='Microsporidia'
and column='pathogen'), but there are many other pathogens in our dataset (light color for
row='pathogen' and column='Microsporidia')

This table might be helpful to inteprete the decission trees and linear models.

In [16]:

```
conditional = []

for category in features:
    conditional.append((X[X[category] == 1.0][features]).mean())

conditional = pd.concat(conditional, axis=1).T
conditional.index = features

sns.heatmap(conditional, cmap=plt.cm.bone_r, square=True);
```

## Differences in proteases aboundance related to a single factor¶

Find significant differences in number of proteases between organism with different lifestyles or taxonomy.
Check single factor at once, eg. compare pathogenic to non-pathogenic fungi, ignoring other differences between the organisms.
Use Mann-Whitney U test to compare the two distributions.

In [17]:

```
significant_difference = pd.DataFrame(data=1.0, index=features, columns=proteases)

for protease in proteases:
    for category in features:
        (val1, tab1), (val2, tab2) = encoded.groupby(category)
        x = tab1[protease]
        y = tab2[protease]
        
        # Ignore groups with less than 20 observation
        if len(x) > 20 and len(y) > 20:
            stat, pvalue = scipy.stats.mannwhitneyu(x, y, alternative='two-sided')
            if pvalue <= 0.05:
                significant_difference.loc[category, protease] = pvalue
```

In [18]:

```
# -log(pvalue)
ax = sns.heatmap(-np.log(significant_difference), cmap=plt.cm.bone_r)
ax.set_title('-log(p)');
```

In [19]:

```
# zoom-in: lifestyle
sns.heatmap(-np.log(significant_difference).loc[lifestyle], cmap=plt.cm.bone_r)
ax.set_title('-log(p)');
```

### Plot distributions of number of proteases for groups with different lifestyles¶

In [20]:

```
def plot_box(protease, category, ax=None):
    global encoded, significant_difference
  
    order = [0.0, 1.0]
    
    if ax is None:
        fig, ax = plt.subplots(figsize=(5.5, 5.5))
    else:
        fig = ax.get_figure()

    sns.boxplot(x=category, y=protease, data=encoded, order=order, ax=ax)
    
    counts = ['(n=%s)' % n for n in data[category].value_counts().loc[order]]
    labels = [' '.join(i) for i in zip(['not %s' % category, category], counts)]
    ax.set_xticklabels(labels)
    ax.set_ylabel('Number of sequences')
    ax.set_xlabel('')
    ax.set_title('%s (p = %.1e)' % (protease, significant_difference.loc[category, protease]))
        
    return fig, ax
```

### Animal¶

In [21]:

```
fig, axs = plt.subplots(ncols=2, nrows=2, figsize=(11, 11))

for protease, ax in zip(['S9', 'S33', 'S54'], axs.flatten()):
    plot_box(protease, 'animal', ax=ax)

axs[-1, -1].set_visible(False)
```

### Pathogen¶

In [22]:

```
fig, axs = plt.subplots(ncols=2, nrows=1, figsize=(11, 5.5))

for protease, ax in zip(['S1', 'S8'], axs):
    plot_box(protease, 'pathogen', ax=ax)
```

### Plant¶

In [23]:

```
fig, axs = plt.subplots(ncols=3, nrows=2, figsize=(15, 11))

for protease, ax in zip(['S9', 'S10', 'S28', 'S33', 'S53'], axs.flatten()):
    plot_box(protease, 'plant', ax=ax)

axs[-1, -1].set_visible(False)
```

## Number of proteases as a function of multiple factors¶

### Decission trees¶

For each protease family build a regression tree predicting number of sequences using information about taxonomy (at subphylum/class level) and lifestyle of an organism.

In [24]:

```
def lifestyle_summary(table):
    """For each lifestyle category compute fractions of organisms in a given table with that lifestyle"""
    lifestyles = table[lifestyle].mean()
    lifestyles = lifestyles[lifestyles > 0.0]
    return ', '.join('%.2f %s' % (value, category) for category, value in lifestyles.items())
```

In [25]:

```
def display_SVG(svg, max_width=600, max_height=500):
    import re
    size_pattern = b'width="%spt" height="%spt"'
    
    width, height = re.findall(size_pattern % (b'([0-9]+)', b'([0-9]+)'), svg)[0]

    height = int(height)
    width = int(width)

    scaling_factor = min(max_width / width, max_height / height)

    resized_svg = svg.replace(size_pattern % tuple(bytes(str(size).encode()) for size in (width, height)),
                              size_pattern % tuple(bytes(str(size*scaling_factor).encode())
                                                   for size in (width, height)))
    display(SVG(resized_svg))
```

In [26]:

```
for protease in proteases:

    Y = encoded[protease]
    
    # Do not build trees for proteases coded by less than 10 organisms
    if (Y > 0).sum() < 10:
        continue
    
    dt = tree.DecisionTreeRegressor(min_samples_leaf=5, max_depth=5, min_impurity_split=0.01)
    dt.fit(X, Y)


    # Print features importance
    print(protease)
    
    print('Feature importance:')
    feature_importances = pd.DataFrame({'importance': dt.feature_importances_}, index=features)
    feature_importances = feature_importances[feature_importances['importance'] > 0]
    feature_importances['lifestyle'] = [lifestyle_summary(data[(data['subphylum'] == i) | (data['class'] == i)])
                                        for i in feature_importances.index]
    feature_importances = feature_importances.sort_values(by='importance', ascending=False)
    display(feature_importances)

    
    # Plot tree
    dot_data = tree.export_graphviz(dt, out_file=None, feature_names=features, filled=True, rounded=True)  
    graph = pydotplus.graph_from_dot_data(dot_data)
    svg = graph.create_svg()
    display_SVG(svg)
    print('\n---------------------------------------------------------------------------------------------------\n')
```

```
S1
Feature importance:
```

|  | importance | lifestyle |
| --- | --- | --- |
| Sordariomycetes | 0.390814 | 0.30 animal, 0.05 fungus, 0.67 plant, 0.86 pathogen, 0.58 saprotroph, 0.02 symbiont, 0.29 soil/dung |
| soil/dung | 0.277544 |  |
| animal | 0.159022 |  |
| pathogen | 0.049887 |  |
| saprotroph | 0.041906 |  |
| Mucoromycotina | 0.034682 | 0.08 animal, 0.08 fungus, 0.08 plant, 0.25 pathogen, 0.92 saprotroph, 0.92 soil/dung |
| Eurotiomycetes | 0.033645 | 0.66 animal, 0.01 fungus, 0.34 plant, 0.60 pathogen, 0.61 saprotroph, 0.02 symbiont, 0.47 soil/dung |
| water | 0.010518 |  |
| plant | 0.001982 |  |

Tree


0

Sordariomycetes <= 0.5
mse = 22.5649
samples = 613
value = 2.9657

1

Eurotiomycetes <= 0.5
mse = 12.4061
samples = 490
value = 2.002

0->1


True

20

animal <= 0.5
mse = 44.5961
samples = 123
value = 6.8049

0->20


False

2

Mucoromycotina <= 0.5
mse = 14.0297
samples = 368
value = 1.6386

1->2


11

saprotroph <= 0.5
mse = 5.9084
samples = 122
value = 3.0984

1->11


3

soil/dung <= 0.5
mse = 13.8286
samples = 356
value = 1.5028

2->3


10

mse = 3.2222
samples = 12
value = 5.6667

2->10


4

water <= 0.5
mse = 2.9173
samples = 260
value = 1.1808

3->4


7

pathogen <= 0.5
mse = 42.3385
samples = 96
value = 2.375

3->7


5

mse = 1.9139
samples = 254
value = 1.1063

4->5


6

mse = 35.2222
samples = 6
value = 4.3333

4->6


8

mse = 2.3071
samples = 70
value = 1.5

7->8


9

mse = 142.5044
samples = 26
value = 4.7308

7->9


12

mse = 9.8711
samples = 48
value = 4.4375

11->12


13

pathogen <= 0.5
mse = 1.4202
samples = 74
value = 2.2297

11->13


14

animal <= 0.5
mse = 1.2387
samples = 46
value = 2.0217

13->14


17

soil/dung <= 0.5
mse = 1.5306
samples = 28
value = 2.5714

13->17


15

mse = 1.3611
samples = 36
value = 2.1667

14->15


16

mse = 0.45
samples = 10
value = 1.5

14->16


18

mse = 1.7633
samples = 13
value = 2.9231

17->18


19

mse = 1.1289
samples = 15
value = 2.2667

17->19


21

saprotroph <= 0.5
mse = 9.058
samples = 86
value = 5.0116

20->21


30

soil/dung <= 0.5
mse = 102.3506
samples = 37
value = 10.973

20->30


22

mse = 2.6556
samples = 33
value = 3.6364

21->22


23

soil/dung <= 0.5
mse = 11.1335
samples = 53
value = 5.8679

21->23


24

pathogen <= 0.5
mse = 11.2441
samples = 35
value = 6.8857

23->24


27

plant <= 0.5
mse = 4.9877
samples = 18
value = 3.8889

23->27


25

mse = 9.25
samples = 8
value = 4.0

24->25


26

mse = 8.6365
samples = 27
value = 7.7407

24->26


28

mse = 1.44
samples = 5
value = 2.6

27->28


29

mse = 5.4675
samples = 13
value = 4.3846

27->29


31

mse = 30.41
samples = 20
value = 5.3

30->31


32

mse = 104.5813
samples = 17
value = 17.6471

30->32

```
---------------------------------------------------------------------------------------------------

S8
Feature importance:
```

|  | importance | lifestyle |
| --- | --- | --- |
| Sordariomycetes | 0.802500 | 0.30 animal, 0.05 fungus, 0.67 plant, 0.86 pathogen, 0.58 saprotroph, 0.02 symbiont, 0.29 soil/dung |
| pathogen | 0.068033 |  |
| Mucoromycotina | 0.043824 | 0.08 animal, 0.08 fungus, 0.08 plant, 0.25 pathogen, 0.92 saprotroph, 0.92 soil/dung |
| saprotroph | 0.023030 |  |
| Saccharomycotina | 0.017361 | 0.49 animal, 0.33 plant, 0.47 pathogen, 0.60 saprotroph, 0.02 symbiont, 0.30 soil/dung, 0.01 water |
| Microsporidia | 0.016292 | 1.00 animal, 1.00 pathogen, 0.08 water |
| animal | 0.010867 |  |
| plant | 0.009913 |  |
| soil/dung | 0.008179 |  |

Tree


0

Sordariomycetes <= 0.5
mse = 106.1038
samples = 613
value = 10.2659

1

Mucoromycotina <= 0.5
mse = 39.6013
samples = 490
value = 6.7469

0->1


True

12

pathogen <= 0.5
mse = 125.1792
samples = 123
value = 24.2846

0->12


False

2

Microsporidia <= 0.5
mse = 36.8004
samples = 478
value = 6.4561

1->2


11

mse = 13.5556
samples = 12
value = 18.3333

1->11


3

Saccharomycotina <= 0.5
mse = 37.4326
samples = 452
value = 6.7279

2->3


10

mse = 2.1967
samples = 26
value = 1.7308

2->10


4

pathogen <= 0.5
mse = 45.5787
samples = 351
value = 7.3732

3->4


7

soil/dung <= 0.5
mse = 2.6458
samples = 101
value = 4.4851

3->7


5

mse = 37.9109
samples = 179
value = 6.0726

4->5


6

mse = 49.966
samples = 172
value = 8.7267

4->6


8

mse = 2.7693
samples = 71
value = 4.8169

7->8


9

mse = 1.4767
samples = 30
value = 3.7

7->9


13

soil/dung <= 0.5
mse = 48.699
samples = 17
value = 14.3529

12->13


16

saprotroph <= 0.5
mse = 119.0887
samples = 106
value = 25.8774

12->16


14

mse = 41.3594
samples = 8
value = 12.125

13->14


15

mse = 46.8889
samples = 9
value = 16.3333

13->15


17

animal <= 0.5
mse = 118.3447
samples = 52
value = 22.9615

16->17


20

soil/dung <= 0.5
mse = 103.7342
samples = 54
value = 28.6852

16->20


18

mse = 121.9008
samples = 33
value = 25.0909

17->18


19

mse = 90.615
samples = 19
value = 19.2632

17->19


21

mse = 86.5306
samples = 28
value = 30.5714

20->21


22

plant <= 0.5
mse = 114.3033
samples = 26
value = 26.6538

20->22


23

mse = 140.1246
samples = 17
value = 29.4118

22->23


24

mse = 24.0247
samples = 9
value = 21.4444

22->24

```
---------------------------------------------------------------------------------------------------

S9
Feature importance:
```

|  | importance | lifestyle |
| --- | --- | --- |
| Microsporidia | 0.348243 | 1.00 animal, 1.00 pathogen, 0.08 water |
| Saccharomycotina | 0.308539 | 0.49 animal, 0.33 plant, 0.47 pathogen, 0.60 saprotroph, 0.02 symbiont, 0.30 soil/dung, 0.01 water |
| Agaricomycetes | 0.167090 | 0.01 animal, 0.97 plant, 0.15 pathogen, 0.80 saprotroph, 0.24 symbiont, 0.17 soil/dung |
| Leotiomycetes | 0.071313 | 0.69 animal, 0.31 plant, 0.31 pathogen, 0.81 saprotroph, 0.06 symbiont, 0.69 soil/dung |
| Tremellomycetes | 0.066279 | 0.66 animal, 0.03 fungus, 0.25 plant, 0.69 pathogen, 1.00 saprotroph, 0.12 soil/dung, 0.09 water |
| pathogen | 0.022580 |  |
| plant | 0.009628 |  |
| saprotroph | 0.004566 |  |
| soil/dung | 0.001755 |  |
| animal | 0.000007 |  |

Tree


0

Saccharomycotina <= 0.5
mse = 5.5884
samples = 613
value = 3.8369

1

Microsporidia <= 0.5
mse = 5.7763
samples = 512
value = 4.2051

0->1


True

16

soil/dung <= 0.5
mse = 0.4645
samples = 101
value = 1.9703

0->16


False

2

Agaricomycetes <= 0.5
mse = 5.1049
samples = 486
value = 4.428

1->2


15

mse = 0.037
samples = 26
value = 0.0385

1->15


3

Leotiomycetes <= 0.5
mse = 4.3454
samples = 415
value = 4.1446

2->3


10

pathogen <= 0.5
mse = 6.3309
samples = 71
value = 6.0845

2->10


4

Tremellomycetes <= 0.5
mse = 4.2088
samples = 383
value = 4.2846

3->4


7

plant <= 0.5
mse = 2.9365
samples = 32
value = 2.4688

3->7


5

mse = 4.2216
samples = 354
value = 4.4237

4->5


6

mse = 0.9322
samples = 29
value = 2.5862

4->6


8

mse = 1.0434
samples = 22
value = 2.0455

7->8


9

mse = 5.84
samples = 10
value = 3.4

7->9


11

saprotroph <= 0.5
mse = 6.0656
samples = 60
value = 6.3667

10->11


14

mse = 4.9752
samples = 11
value = 4.5455

10->14


12

mse = 2.1684
samples = 14
value = 5.7857

11->12


13

mse = 7.1177
samples = 46
value = 6.5435

11->13


17

saprotroph <= 0.5
mse = 0.319
samples = 71
value = 2.0704

16->17


22

plant <= 0.5
mse = 0.7289
samples = 30
value = 1.7333

16->22


18

mse = 0.1368
samples = 41
value = 2.0976

17->18


19

animal <= 0.5
mse = 0.5656
samples = 30
value = 2.0333

17->19


20

mse = 0.7372
samples = 23
value = 2.0435

19->20


21

mse = 0.0
samples = 7
value = 2.0

19->21


23

mse = 0.8889
samples = 24
value = 1.6667

22->23


24

mse = 0.0
samples = 6
value = 2.0

22->24

```
---------------------------------------------------------------------------------------------------

S10
Feature importance:
```

|  | importance | lifestyle |
| --- | --- | --- |
| Saccharomycotina | 0.358280 | 0.49 animal, 0.33 plant, 0.47 pathogen, 0.60 saprotroph, 0.02 symbiont, 0.30 soil/dung, 0.01 water |
| Microsporidia | 0.326000 | 1.00 animal, 1.00 pathogen, 0.08 water |
| Agaricomycetes | 0.127844 | 0.01 animal, 0.97 plant, 0.15 pathogen, 0.80 saprotroph, 0.24 symbiont, 0.17 soil/dung |
| Tremellomycetes | 0.075602 | 0.66 animal, 0.03 fungus, 0.25 plant, 0.69 pathogen, 1.00 saprotroph, 0.12 soil/dung, 0.09 water |
| Taphrinomycotina | 0.045025 | 0.71 animal, 0.14 fungus, 0.43 plant, 0.60 pathogen, 0.40 saprotroph, 0.14 soil/dung |
| symbiont | 0.038939 |  |
| pathogen | 0.024458 |  |
| animal | 0.003404 |  |
| saprotroph | 0.000251 |  |
| plant | 0.000144 |  |
| soil/dung | 0.000053 |  |

Tree


0

Saccharomycotina <= 0.5
mse = 20.5992
samples = 613
value = 7.3605

1

Microsporidia <= 0.5
mse = 20.4614
samples = 512
value = 8.1641

0->1


True

16

animal <= 0.5
mse = 1.4324
samples = 101
value = 3.2871

0->16


False

2

Agaricomycetes <= 0.5
mse = 17.7995
samples = 486
value = 8.6008

1->2


15

mse = 0.0
samples = 26
value = 0.0

1->15


3

Tremellomycetes <= 0.5
mse = 13.0047
samples = 415
value = 8.0988

2->3


10

symbiont <= 0.5
mse = 35.7417
samples = 71
value = 11.5352

2->10


4

Taphrinomycotina <= 0.5
mse = 12.7734
samples = 386
value = 8.3756

3->4


7

pathogen <= 0.5
mse = 1.4839
samples = 29
value = 4.4138

3->7


5

mse = 12.3131
samples = 379
value = 8.4855

4->5


6

mse = 1.6735
samples = 7
value = 2.4286

4->6


8

mse = 1.3333
samples = 9
value = 3.6667

7->8


9

mse = 1.1875
samples = 20
value = 4.75

7->9


11

pathogen <= 0.5
mse = 39.0274
samples = 54
value = 12.5185

10->11


14

mse = 12.4775
samples = 17
value = 8.4118

10->14


12

mse = 39.5133
samples = 43
value = 13.3023

11->12


13

mse = 25.3388
samples = 11
value = 9.4545

11->13


17

plant <= 0.5
mse = 1.1165
samples = 52
value = 2.8654

16->17


22

saprotroph <= 0.5
mse = 1.3786
samples = 49
value = 3.7347

16->22


18

mse = 1.0612
samples = 21
value = 2.7143

17->18


19

soil/dung <= 0.5
mse = 1.128
samples = 31
value = 2.9677

17->19


20

mse = 1.3536
samples = 25
value = 2.92

19->20


21

mse = 0.1389
samples = 6
value = 3.1667

19->21


23

mse = 1.3268
samples = 39
value = 3.8205

22->23


24

mse = 1.44
samples = 10
value = 3.4

22->24

```
---------------------------------------------------------------------------------------------------

S12
Feature importance:
```

|  | importance | lifestyle |
| --- | --- | --- |
| Sordariomycetes | 0.375495 | 0.30 animal, 0.05 fungus, 0.67 plant, 0.86 pathogen, 0.58 saprotroph, 0.02 symbiont, 0.29 soil/dung |
| Eurotiomycetes | 0.186917 | 0.66 animal, 0.01 fungus, 0.34 plant, 0.60 pathogen, 0.61 saprotroph, 0.02 symbiont, 0.47 soil/dung |
| Leotiomycetes | 0.122958 | 0.69 animal, 0.31 plant, 0.31 pathogen, 0.81 saprotroph, 0.06 symbiont, 0.69 soil/dung |
| Dothideomycetes | 0.110008 | 0.03 animal, 0.95 plant, 0.59 pathogen, 0.64 saprotroph, 0.05 symbiont, 0.54 soil/dung |
| pathogen | 0.070281 |  |
| Agaricomycetes | 0.042444 | 0.01 animal, 0.97 plant, 0.15 pathogen, 0.80 saprotroph, 0.24 symbiont, 0.17 soil/dung |
| soil/dung | 0.034603 |  |
| saprotroph | 0.033941 |  |
| plant | 0.012896 |  |
| animal | 0.010458 |  |

Tree


0

Sordariomycetes <= 0.5
mse = 45.058
samples = 613
value = 6.4339

1

Eurotiomycetes <= 0.5
mse = 33.361
samples = 490
value = 4.9082

0->1


True

28

pathogen <= 0.5
mse = 45.4368
samples = 123
value = 12.5122

0->28


False

2

Leotiomycetes <= 0.5
mse = 27.8309
samples = 368
value = 3.5245

1->2


15

soil/dung <= 0.5
mse = 26.8457
samples = 122
value = 9.082

1->15


3

Dothideomycetes <= 0.5
mse = 19.7778
samples = 336
value = 2.8304

2->3


10

pathogen <= 0.5
mse = 54.2148
samples = 32
value = 10.8125

2->10


4

Agaricomycetes <= 0.5
mse = 14.9725
samples = 297
value = 2.0236

3->4


7

pathogen <= 0.5
mse = 13.666
samples = 39
value = 8.9744

3->7


5

mse = 7.655
samples = 226
value = 1.1991

4->5


6

mse = 29.214
samples = 71
value = 4.6479

4->6


8

mse = 21.6094
samples = 16
value = 10.125

7->8


9

mse = 6.5784
samples = 23
value = 8.1739

7->9


11

soil/dung <= 0.5
mse = 45.5372
samples = 22
value = 13.0909

10->11


14

mse = 36.76
samples = 10
value = 5.8

10->14


12

mse = 147.5556
samples = 6
value = 11.3333

11->12


13

mse = 5.6875
samples = 16
value = 13.75

11->13


16

pathogen <= 0.5
mse = 24.4151
samples = 65
value = 7.7846

15->16


21

plant <= 0.5
mse = 25.5094
samples = 57
value = 10.5614

15->21


17

mse = 19.8056
samples = 6
value = 9.8333

16->17


18

saprotroph <= 0.5
mse = 24.4137
samples = 59
value = 7.5763

16->18


19

mse = 24.233
samples = 46
value = 7.6304

18->19


20

mse = 25.0059
samples = 13
value = 7.3846

18->20


22

pathogen <= 0.5
mse = 42.5806
samples = 22
value = 11.3182

21->22


25

animal <= 0.5
mse = 14.1927
samples = 35
value = 10.0857

21->25


23

mse = 26.6582
samples = 14
value = 10.3571

22->23


24

mse = 66.0
samples = 8
value = 13.0

22->24


26

mse = 13.4897
samples = 22
value = 10.6818

25->26


27

mse = 13.7633
samples = 13
value = 9.0769

25->27


29

soil/dung <= 0.5
mse = 11.0588
samples = 17
value = 7.0

28->29


32

saprotroph <= 0.5
mse = 45.2958
samples = 106
value = 13.3962

28->32


30

mse = 14.4844
samples = 8
value = 7.375

29->30


31

mse = 7.7778
samples = 9
value = 6.6667

29->31


33

plant <= 0.5
mse = 50.3225
samples = 52
value = 11.1538

32->33


36

soil/dung <= 0.5
mse = 30.9506
samples = 54
value = 15.5556

32->36


34

mse = 56.9977
samples = 21
value = 13.381

33->34


35

mse = 40.1644
samples = 31
value = 9.6452

33->35


37

mse = 23.5753
samples = 28
value = 17.6786

36->37


38

animal <= 0.5
mse = 28.8121
samples = 26
value = 13.2692

36->38


39

mse = 17.8765
samples = 9
value = 10.1111

38->39


40

mse = 26.526
samples = 17
value = 14.9412

38->40

```
---------------------------------------------------------------------------------------------------

S14
Feature importance:
```

|  | importance | lifestyle |
| --- | --- | --- |
| Saccharomycotina | 0.581963 | 0.49 animal, 0.33 plant, 0.47 pathogen, 0.60 saprotroph, 0.02 symbiont, 0.30 soil/dung, 0.01 water |
| Microsporidia | 0.189458 | 1.00 animal, 1.00 pathogen, 0.08 water |
| Ustilaginomycotina | 0.155795 | 0.28 animal, 0.78 plant, 0.72 pathogen, 0.33 saprotroph |
| saprotroph | 0.033559 |  |
| Sordariomycetes | 0.019910 | 0.30 animal, 0.05 fungus, 0.67 plant, 0.86 pathogen, 0.58 saprotroph, 0.02 symbiont, 0.29 soil/dung |
| Pucciniomycotina | 0.015344 | 0.92 plant, 0.67 pathogen, 0.25 saprotroph, 0.08 symbiont, 0.08 soil/dung, 0.08 water |
| plant | 0.002985 |  |
| soil/dung | 0.000985 |  |

Tree


0

Saccharomycotina <= 0.5
mse = 0.3396
samples = 613
value = 0.7651

1

Microsporidia <= 0.5
mse = 0.2748
samples = 512
value = 0.9082

0->1


True

12

plant <= 0.5
mse = 0.038
samples = 101
value = 0.0396

0->12


False

2

Ustilaginomycotina <= 0.5
mse = 0.2449
samples = 486
value = 0.9547

1->2


11

mse = 0.037
samples = 26
value = 0.0385

1->11


3

Sordariomycetes <= 0.5
mse = 0.2179
samples = 468
value = 0.9915

2->3


10

mse = 0.0
samples = 18
value = 0.0

2->10


4

Pucciniomycotina <= 0.5
mse = 0.1048
samples = 345
value = 0.9507

3->4


7

saprotroph <= 0.5
mse = 0.5173
samples = 123
value = 1.1057

3->7


5

mse = 0.0888
samples = 333
value = 0.964

4->5


6

mse = 0.4097
samples = 12
value = 0.5833

4->6


8

mse = 0.0869
samples = 52
value = 0.9038

7->8


9

mse = 0.7808
samples = 71
value = 1.2535

7->9


13

mse = 0.0
samples = 68
value = 0.0

12->13


14

soil/dung <= 0.5
mse = 0.1065
samples = 33
value = 0.1212

12->14


15

mse = 0.1262
samples = 27
value = 0.1481

14->15


16

mse = 0.0
samples = 6
value = 0.0

14->16

```
---------------------------------------------------------------------------------------------------

S15
Feature importance:
```

|  | importance | lifestyle |
| --- | --- | --- |
| Sordariomycetes | 0.280253 | 0.30 animal, 0.05 fungus, 0.67 plant, 0.86 pathogen, 0.58 saprotroph, 0.02 symbiont, 0.29 soil/dung |
| saprotroph | 0.188536 |  |
| plant | 0.167996 |  |
| soil/dung | 0.159683 |  |
| pathogen | 0.111440 |  |
| Eurotiomycetes | 0.046379 | 0.66 animal, 0.01 fungus, 0.34 plant, 0.60 pathogen, 0.61 saprotroph, 0.02 symbiont, 0.47 soil/dung |
| Dothideomycetes | 0.029602 | 0.03 animal, 0.95 plant, 0.59 pathogen, 0.64 saprotroph, 0.05 symbiont, 0.54 soil/dung |
| animal | 0.008746 |  |
| Saccharomycotina | 0.003296 | 0.49 animal, 0.33 plant, 0.47 pathogen, 0.60 saprotroph, 0.02 symbiont, 0.30 soil/dung, 0.01 water |
| fungus | 0.002991 |  |
| symbiont | 0.001078 |  |

Tree


0

Sordariomycetes <= 0.5
mse = 0.5078
samples = 613
value = 0.2512

1

Eurotiomycetes <= 0.5
mse = 0.2008
samples = 490
value = 0.1327

0->1


True

28

plant <= 0.5
mse = 1.452
samples = 123
value = 0.7236

0->28


False

2

Dothideomycetes <= 0.5
mse = 0.0928
samples = 368
value = 0.0707

1->2


15

soil/dung <= 0.5
mse = 0.4798
samples = 122
value = 0.3197

1->15


3

Saccharomycotina <= 0.5
mse = 0.0473
samples = 329
value = 0.0365

2->3


10

pathogen <= 0.5
mse = 0.384
samples = 39
value = 0.359

2->10


4

symbiont <= 0.5
mse = 0.0218
samples = 228
value = 0.0132

3->4


7

saprotroph <= 0.5
mse = 0.101
samples = 101
value = 0.0891

3->7


5

mse = 0.0049
samples = 204
value = 0.0049

4->5


6

mse = 0.1597
samples = 24
value = 0.0833

4->6


8

mse = 0.0
samples = 41
value = 0.0

7->8


9

mse = 0.1608
samples = 60
value = 0.15

7->9


11

mse = 0.4961
samples = 16
value = 0.4375

10->11


12

saprotroph <= 0.5
mse = 0.2987
samples = 23
value = 0.3043

10->12


13

mse = 0.4097
samples = 12
value = 0.4167

12->13


14

mse = 0.1488
samples = 11
value = 0.1818

12->14


16

plant <= 0.5
mse = 0.3394
samples = 65
value = 0.2462

15->16


21

pathogen <= 0.5
mse = 0.6267
samples = 57
value = 0.4035

15->21


17

saprotroph <= 0.5
mse = 0.3641
samples = 58
value = 0.2586

16->17


20

mse = 0.1224
samples = 7
value = 0.1429

16->20


18

mse = 0.4102
samples = 46
value = 0.2609

17->18


19

mse = 0.1875
samples = 12
value = 0.25

17->19


22

animal <= 0.5
mse = 0.2517
samples = 42
value = 0.2857

21->22


25

plant <= 0.5
mse = 1.5289
samples = 15
value = 0.7333

21->25


23

mse = 0.2969
samples = 32
value = 0.375

22->23


24

mse = 0.0
samples = 10
value = 0.0

22->24


26

mse = 2.6875
samples = 8
value = 0.75

25->26


27

mse = 0.2041
samples = 7
value = 0.7143

25->27


29

fungus <= 0.5
mse = 0.1737
samples = 41
value = 0.1463

28->29


34

soil/dung <= 0.5
mse = 1.8413
samples = 82
value = 1.0122

28->34


30

soil/dung <= 0.5
mse = 0.1543
samples = 36
value = 0.1111

29->30


33

mse = 0.24
samples = 5
value = 0.4

29->33


31

mse = 0.0942
samples = 19
value = 0.1053

30->31


32

mse = 0.2215
samples = 17
value = 0.1176

30->32


35

pathogen <= 0.5
mse = 1.9728
samples = 67
value = 1.2388

34->35


40

mse = 0.0
samples = 15
value = 0.0

34->40


36

mse = 0.1094
samples = 8
value = 0.125

35->36


37

saprotroph <= 0.5
mse = 2.0345
samples = 59
value = 1.3898

35->37


38

mse = 1.64
samples = 31
value = 0.8065

37->38


39

mse = 1.6773
samples = 28
value = 2.0357

37->39

```
---------------------------------------------------------------------------------------------------

S16
Feature importance:
```

|  | importance | lifestyle |
| --- | --- | --- |
| Mucoromycotina | 0.477143 | 0.08 animal, 0.08 fungus, 0.08 plant, 0.25 pathogen, 0.92 saprotroph, 0.92 soil/dung |
| Microsporidia | 0.333634 | 1.00 animal, 1.00 pathogen, 0.08 water |
| Eurotiomycetes | 0.065493 | 0.66 animal, 0.01 fungus, 0.34 plant, 0.60 pathogen, 0.61 saprotroph, 0.02 symbiont, 0.47 soil/dung |
| saprotroph | 0.044145 |  |
| plant | 0.035617 |  |
| Tremellomycetes | 0.029070 | 0.66 animal, 0.03 fungus, 0.25 plant, 0.69 pathogen, 1.00 saprotroph, 0.12 soil/dung, 0.09 water |
| Saccharomycotina | 0.014899 | 0.49 animal, 0.33 plant, 0.47 pathogen, 0.60 saprotroph, 0.02 symbiont, 0.30 soil/dung, 0.01 water |

Tree


0

Mucoromycotina <= 0.5
mse = 0.831
samples = 613
value = 1.9233

1

Microsporidia <= 0.5
mse = 0.584
samples = 601
value = 1.8586

0->1


True

16

mse = 2.4722
samples = 12
value = 5.1667

0->16


False

2

Eurotiomycetes <= 0.5
mse = 0.4522
samples = 575
value = 1.9409

1->2


15

mse = 0.037
samples = 26
value = 0.0385

1->15


3

plant <= 0.5
mse = 0.3395
samples = 453
value = 1.8499

2->3


10

saprotroph <= 0.5
mse = 0.7256
samples = 122
value = 2.2787

2->10


4

Tremellomycetes <= 0.5
mse = 0.3816
samples = 175
value = 1.6686

3->4


7

Saccharomycotina <= 0.5
mse = 0.2793
samples = 278
value = 1.964

3->7


5

mse = 0.3709
samples = 154
value = 1.7468

4->5


6

mse = 0.0862
samples = 21
value = 1.0952

4->6


8

mse = 0.2367
samples = 245
value = 2.0082

7->8


9

mse = 0.4738
samples = 33
value = 1.6364

7->9


11

mse = 1.5139
samples = 48
value = 2.6667

10->11


12

plant <= 0.5
mse = 0.0533
samples = 74
value = 2.027

10->12


13

mse = 0.0804
samples = 34
value = 2.0882

12->13


14

mse = 0.0244
samples = 40
value = 1.975

12->14

```
---------------------------------------------------------------------------------------------------

S26
Feature importance:
```

|  | importance | lifestyle |
| --- | --- | --- |
| Microsporidia | 0.813490 | 1.00 animal, 1.00 pathogen, 0.08 water |
| water | 0.083980 |  |
| Tremellomycetes | 0.041183 | 0.66 animal, 0.03 fungus, 0.25 plant, 0.69 pathogen, 1.00 saprotroph, 0.12 soil/dung, 0.09 water |
| Ustilaginomycotina | 0.022622 | 0.28 animal, 0.78 plant, 0.72 pathogen, 0.33 saprotroph |
| Dothideomycetes | 0.018025 | 0.03 animal, 0.95 plant, 0.59 pathogen, 0.64 saprotroph, 0.05 symbiont, 0.54 soil/dung |
| animal | 0.013883 |  |
| saprotroph | 0.006818 |  |

Tree


0

Microsporidia <= 0.5
mse = 0.7226
samples = 613
value = 2.8238

1

water <= 0.5
mse = 0.6069
samples = 587
value = 2.9012

0->1


True

14

mse = 0.1479
samples = 26
value = 1.0769

0->14


False

2

Tremellomycetes <= 0.5
mse = 0.5685
samples = 580
value = 2.8879

1->2


13

mse = 2.5714
samples = 7
value = 4.0

1->13


3

Ustilaginomycotina <= 0.5
mse = 0.5704
samples = 551
value = 2.9074

2->3


10

animal <= 0.5
mse = 0.3876
samples = 29
value = 2.5172

2->10


4

Dothideomycetes <= 0.5
mse = 0.5732
samples = 533
value = 2.9193

3->4


7

saprotroph <= 0.5
mse = 0.358
samples = 18
value = 2.5556

3->7


5

mse = 0.5938
samples = 494
value = 2.9028

4->5


6

mse = 0.2656
samples = 39
value = 3.1282

4->6


8

mse = 0.4097
samples = 12
value = 2.4167

7->8


9

mse = 0.1389
samples = 6
value = 2.8333

7->9


11

mse = 0.1094
samples = 8
value = 2.875

10->11


12

mse = 0.4263
samples = 21
value = 2.381

10->12

```
---------------------------------------------------------------------------------------------------

S28
Feature importance:
```

|  | importance | lifestyle |
| --- | --- | --- |
| Saccharomycotina | 0.599032 | 0.49 animal, 0.33 plant, 0.47 pathogen, 0.60 saprotroph, 0.02 symbiont, 0.30 soil/dung, 0.01 water |
| Microsporidia | 0.194503 | 1.00 animal, 1.00 pathogen, 0.08 water |
| Tremellomycetes | 0.078186 | 0.66 animal, 0.03 fungus, 0.25 plant, 0.69 pathogen, 1.00 saprotroph, 0.12 soil/dung, 0.09 water |
| Ustilaginomycotina | 0.069764 | 0.28 animal, 0.78 plant, 0.72 pathogen, 0.33 saprotroph |
| Taphrinomycotina | 0.056978 | 0.71 animal, 0.14 fungus, 0.43 plant, 0.60 pathogen, 0.40 saprotroph, 0.14 soil/dung |
| pathogen | 0.000836 |  |
| animal | 0.000701 |  |

Tree


0

Saccharomycotina <= 0.5
mse = 3.6749
samples = 613
value = 2.0212

1

Microsporidia <= 0.5
mse = 3.435
samples = 512
value = 2.4199

0->1


True

14

mse = 0.0
samples = 101
value = 0.0

0->14


False

2

Tremellomycetes <= 0.5
mse = 3.2887
samples = 486
value = 2.5494

1->2


13

mse = 0.0
samples = 26
value = 0.0

1->13


3

Ustilaginomycotina <= 0.5
mse = 3.3417
samples = 457
value = 2.6411

2->3


10

pathogen <= 0.5
mse = 0.2307
samples = 29
value = 1.1034

2->10


4

Taphrinomycotina <= 0.5
mse = 3.3436
samples = 439
value = 2.713

3->4


7

animal <= 0.5
mse = 0.0988
samples = 18
value = 0.8889

3->7


5

mse = 3.287
samples = 432
value = 2.7546

4->5


6

mse = 0.1224
samples = 7
value = 0.1429

4->6


8

mse = 0.0
samples = 13
value = 1.0

7->8


9

mse = 0.24
samples = 5
value = 0.6

7->9


11

mse = 0.4444
samples = 9
value = 1.3333

10->11


12

mse = 0.1
samples = 20
value = 1.0

10->12

```
---------------------------------------------------------------------------------------------------

S33
Feature importance:
```

|  | importance | lifestyle |
| --- | --- | --- |
| Agaricomycetes | 0.663519 | 0.01 animal, 0.97 plant, 0.15 pathogen, 0.80 saprotroph, 0.24 symbiont, 0.17 soil/dung |
| symbiont | 0.113320 |  |
| Saccharomycotina | 0.087484 | 0.49 animal, 0.33 plant, 0.47 pathogen, 0.60 saprotroph, 0.02 symbiont, 0.30 soil/dung, 0.01 water |
| pathogen | 0.086666 |  |
| Microsporidia | 0.029970 | 1.00 animal, 1.00 pathogen, 0.08 water |
| Mucoromycotina | 0.014016 | 0.08 animal, 0.08 fungus, 0.08 plant, 0.25 pathogen, 0.92 saprotroph, 0.92 soil/dung |
| soil/dung | 0.005024 |  |

Tree


0

Agaricomycetes <= 0.5
mse = 3.9018
samples = 613
value = 1.2463

1

Saccharomycotina <= 0.5
mse = 1.6382
samples = 542
value = 0.8635

0->1


True

10

pathogen <= 0.5
mse = 11.5207
samples = 71
value = 4.169

0->10


False

2

Microsporidia <= 0.5
mse = 1.806
samples = 441
value = 1.059

1->2


9

mse = 0.0098
samples = 101
value = 0.0099

1->9


3

symbiont <= 0.5
mse = 1.8445
samples = 415
value = 1.1253

2->3


8

mse = 0.0
samples = 26
value = 0.0

2->8


4

Mucoromycotina <= 0.5
mse = 1.2725
samples = 401
value = 1.0823

3->4


7

mse = 16.6582
samples = 14
value = 2.3571

3->7


5

mse = 1.2745
samples = 389
value = 1.1157

4->5


6

mse = 0.0
samples = 12
value = 0.0

4->6


11

symbiont <= 0.5
mse = 11.6608
samples = 60
value = 4.65

10->11


16

mse = 2.6116
samples = 11
value = 1.5455

10->16


12

soil/dung <= 0.5
mse = 13.5024
samples = 43
value = 5.4419

11->12


15

mse = 1.4048
samples = 17
value = 2.6471

11->15


13

mse = 13.4792
samples = 38
value = 5.3158

12->13


14

mse = 12.64
samples = 5
value = 6.4

12->14

```
---------------------------------------------------------------------------------------------------

S41
Feature importance:
```

|  | importance | lifestyle |
| --- | --- | --- |
| water | 0.322458 |  |
| Dothideomycetes | 0.322321 | 0.03 animal, 0.95 plant, 0.59 pathogen, 0.64 saprotroph, 0.05 symbiont, 0.54 soil/dung |
| Saccharomycotina | 0.189290 | 0.49 animal, 0.33 plant, 0.47 pathogen, 0.60 saprotroph, 0.02 symbiont, 0.30 soil/dung, 0.01 water |
| Sordariomycetes | 0.073350 | 0.30 animal, 0.05 fungus, 0.67 plant, 0.86 pathogen, 0.58 saprotroph, 0.02 symbiont, 0.29 soil/dung |
| Tremellomycetes | 0.049088 | 0.66 animal, 0.03 fungus, 0.25 plant, 0.69 pathogen, 1.00 saprotroph, 0.12 soil/dung, 0.09 water |
| pathogen | 0.042931 |  |
| soil/dung | 0.000561 |  |

Tree


0

Dothideomycetes <= 0.5
mse = 7.0026
samples = 613
value = 1.5661

1

water <= 0.5
mse = 6.4533
samples = 574
value = 1.3728

0->1


True

12

soil/dung <= 0.5
mse = 6.4471
samples = 39
value = 4.4103

0->12


False

2

Saccharomycotina <= 0.5
mse = 2.9822
samples = 565
value = 1.2761

1->2


11

mse = 186.9136
samples = 9
value = 7.4444

1->11


3

Sordariomycetes <= 0.5
mse = 3.198
samples = 465
value = 1.5505

2->3


10

mse = 0.0
samples = 100
value = 0.0

2->10


4

Tremellomycetes <= 0.5
mse = 2.78
samples = 342
value = 1.307

3->4


7

pathogen <= 0.5
mse = 3.7368
samples = 123
value = 2.2276

3->7


5

mse = 2.8706
samples = 313
value = 1.4249

4->5


6

mse = 0.0333
samples = 29
value = 0.0345

4->6


8

mse = 1.3979
samples = 17
value = 1.1176

7->8


9

mse = 3.8826
samples = 106
value = 2.4057

7->9


13

mse = 5.8673
samples = 18
value = 4.2778

12->13


14

pathogen <= 0.5
mse = 6.9161
samples = 21
value = 4.5238

12->14


15

mse = 9.7222
samples = 12
value = 3.6667

14->15


16

mse = 0.8889
samples = 9
value = 5.6667

14->16

```
---------------------------------------------------------------------------------------------------

S49
Feature importance:
```

|  | importance | lifestyle |
| --- | --- | --- |
| Ustilaginomycotina | 0.459605 | 0.28 animal, 0.78 plant, 0.72 pathogen, 0.33 saprotroph |
| Pucciniomycotina | 0.453650 | 0.92 plant, 0.67 pathogen, 0.25 saprotroph, 0.08 symbiont, 0.08 soil/dung, 0.08 water |
| water | 0.049815 |  |
| saprotroph | 0.021112 |  |
| plant | 0.010112 |  |
| soil/dung | 0.005707 |  |

Tree


0

Ustilaginomycotina <= 0.5
mse = 0.0654
samples = 613
value = 0.0555

1

Pucciniomycotina <= 0.5
mse = 0.0459
samples = 595
value = 0.0336

0->1


True

10

saprotroph <= 0.5
mse = 0.1728
samples = 18
value = 0.7778

0->10


False

2

water <= 0.5
mse = 0.0289
samples = 583
value = 0.0154

1->2


9

mse = 0.0764
samples = 12
value = 0.9167

1->9


3

soil/dung <= 0.5
mse = 0.0208
samples = 575
value = 0.0104

2->3


8

mse = 0.4844
samples = 8
value = 0.375

2->8


4

mse = 0.0
samples = 378
value = 0.0

3->4


5

plant <= 0.5
mse = 0.06
samples = 197
value = 0.0305

3->5


6

mse = 0.0
samples = 106
value = 0.0

5->6


7

mse = 0.1275
samples = 91
value = 0.0659

5->7


11

mse = 0.2222
samples = 12
value = 0.6667

10->11


12

mse = 0.0
samples = 6
value = 1.0

10->12

```
---------------------------------------------------------------------------------------------------

S51
Feature importance:
```

|  | importance | lifestyle |
| --- | --- | --- |
| Dothideomycetes | 0.451363 | 0.03 animal, 0.95 plant, 0.59 pathogen, 0.64 saprotroph, 0.05 symbiont, 0.54 soil/dung |
| fungus | 0.245118 |  |
| Sordariomycetes | 0.188238 | 0.30 animal, 0.05 fungus, 0.67 plant, 0.86 pathogen, 0.58 saprotroph, 0.02 symbiont, 0.29 soil/dung |
| pathogen | 0.052129 |  |
| saprotroph | 0.025944 |  |
| soil/dung | 0.024075 |  |
| animal | 0.013133 |  |

Tree


0

Dothideomycetes <= 0.5
mse = 0.0436
samples = 613
value = 0.0457

1

Sordariomycetes <= 0.5
mse = 0.0254
samples = 574
value = 0.0261

0->1


True

12

saprotroph <= 0.5
mse = 0.2222
samples = 39
value = 0.3333

0->12


False

2

mse = 0.0
samples = 451
value = 0.0

1->2


3

fungus <= 0.5
mse = 0.1071
samples = 123
value = 0.122

1->3


4

pathogen <= 0.5
mse = 0.0852
samples = 117
value = 0.094

3->4


11

mse = 0.2222
samples = 6
value = 0.6667

3->11


5

soil/dung <= 0.5
mse = 0.1799
samples = 17
value = 0.2353

4->5


8

animal <= 0.5
mse = 0.0651
samples = 100
value = 0.07

4->8


6

mse = 0.1094
samples = 8
value = 0.125

5->6


7

mse = 0.2222
samples = 9
value = 0.3333

5->7


9

mse = 0.085
samples = 64
value = 0.0938

8->9


10

mse = 0.027
samples = 36
value = 0.0278

8->10


13

mse = 0.2449
samples = 14
value = 0.4286

12->13


14

pathogen <= 0.5
mse = 0.2016
samples = 25
value = 0.28

12->14


15

mse = 0.2041
samples = 14
value = 0.2857

14->15


16

mse = 0.1983
samples = 11
value = 0.2727

14->16

```
---------------------------------------------------------------------------------------------------

S53
Feature importance:
```

|  | importance | lifestyle |
| --- | --- | --- |
| Agaricomycetes | 0.368513 | 0.01 animal, 0.97 plant, 0.15 pathogen, 0.80 saprotroph, 0.24 symbiont, 0.17 soil/dung |
| Saccharomycotina | 0.167849 | 0.49 animal, 0.33 plant, 0.47 pathogen, 0.60 saprotroph, 0.02 symbiont, 0.30 soil/dung, 0.01 water |
| Sordariomycetes | 0.095839 | 0.30 animal, 0.05 fungus, 0.67 plant, 0.86 pathogen, 0.58 saprotroph, 0.02 symbiont, 0.29 soil/dung |
| saprotroph | 0.089000 |  |
| pathogen | 0.071219 |  |
| Eurotiomycetes | 0.058197 | 0.66 animal, 0.01 fungus, 0.34 plant, 0.60 pathogen, 0.61 saprotroph, 0.02 symbiont, 0.47 soil/dung |
| animal | 0.057269 |  |
| plant | 0.051345 |  |
| Tremellomycetes | 0.021780 | 0.66 animal, 0.03 fungus, 0.25 plant, 0.69 pathogen, 1.00 saprotroph, 0.12 soil/dung, 0.09 water |
| fungus | 0.014338 |  |
| soil/dung | 0.004651 |  |

Tree


0

Agaricomycetes <= 0.5
mse = 13.1042
samples = 613
value = 2.9086

1

Saccharomycotina <= 0.5
mse = 7.7974
samples = 542
value = 2.3432

0->1


True

18

saprotroph <= 0.5
mse = 32.5408
samples = 71
value = 7.2254

0->18


False

2

Sordariomycetes <= 0.5
mse = 8.0377
samples = 441
value = 2.8798

1->2


17

mse = 0.0
samples = 101
value = 0.0

1->17


3

plant <= 0.5
mse = 5.1202
samples = 318
value = 2.2956

2->3


10

animal <= 0.5
mse = 12.4168
samples = 123
value = 4.3902

2->10


4

Eurotiomycetes <= 0.5
mse = 2.761
samples = 181
value = 1.5912

3->4


7

Tremellomycetes <= 0.5
mse = 6.7152
samples = 137
value = 3.2263

3->7


5

mse = 1.5118
samples = 101
value = 0.5743

4->5


6

mse = 1.3844
samples = 80
value = 2.875

4->6


8

mse = 6.4461
samples = 129
value = 3.4264

7->8


9

mse = 0.0
samples = 8
value = 0.0

7->9


11

fungus <= 0.5
mse = 3.6917
samples = 86
value = 3.4884

10->11


14

saprotroph <= 0.5
mse = 26.412
samples = 37
value = 6.4865

10->14


12

mse = 2.1539
samples = 81
value = 3.284

11->12


13

mse = 16.96
samples = 5
value = 6.8

11->13


15

mse = 45.7784
samples = 19
value = 6.8947

14->15


16

mse = 5.608
samples = 18
value = 6.0556

14->16


19

mse = 5.3469
samples = 14
value = 2.7143

18->19


20

pathogen <= 0.5
mse = 32.9942
samples = 57
value = 8.3333

18->20


21

soil/dung <= 0.5
mse = 30.811
samples = 46
value = 9.4348

20->21


24

mse = 15.8347
samples = 11
value = 3.7273

20->24


22

mse = 27.0541
samples = 41
value = 9.6585

21->22


23

mse = 57.84
samples = 5
value = 7.6

21->23

```
---------------------------------------------------------------------------------------------------

S54
Feature importance:
```

|  | importance | lifestyle |
| --- | --- | --- |
| Microsporidia | 0.501216 | 1.00 animal, 1.00 pathogen, 0.08 water |
| Mucoromycotina | 0.177608 | 0.08 animal, 0.08 fungus, 0.08 plant, 0.25 pathogen, 0.92 saprotroph, 0.92 soil/dung |
| Dothideomycetes | 0.129993 | 0.03 animal, 0.95 plant, 0.59 pathogen, 0.64 saprotroph, 0.05 symbiont, 0.54 soil/dung |
| water | 0.093870 |  |
| Saccharomycotina | 0.090099 | 0.49 animal, 0.33 plant, 0.47 pathogen, 0.60 saprotroph, 0.02 symbiont, 0.30 soil/dung, 0.01 water |
| soil/dung | 0.006390 |  |
| pathogen | 0.000824 |  |

Tree


0

Microsporidia <= 0.5
mse = 2.3032
samples = 613
value = 3.0587

1

Mucoromycotina <= 0.5
mse = 1.9909
samples = 587
value = 3.1908

0->1


True

14

mse = 0.071
samples = 26
value = 0.0769

0->14


False

2

Dothideomycetes <= 0.5
mse = 1.8216
samples = 575
value = 3.1357

1->2


13

mse = 2.9722
samples = 12
value = 5.8333

1->13


3

water <= 0.5
mse = 1.7609
samples = 536
value = 3.0466

2->3


8

soil/dung <= 0.5
mse = 1.0506
samples = 39
value = 4.359

2->8


4

Saccharomycotina <= 0.5
mse = 1.5102
samples = 529
value = 3.0132

3->4


7

mse = 14.2449
samples = 7
value = 5.5714

3->7


5

mse = 1.6344
samples = 429
value = 3.1515

4->5


6

mse = 0.5436
samples = 100
value = 2.42

4->6


9

mse = 1.3858
samples = 18
value = 4.0556

8->9


10

pathogen <= 0.5
mse = 0.6168
samples = 21
value = 4.619

8->10


11

mse = 0.9167
samples = 12
value = 4.5

10->11


12

mse = 0.1728
samples = 9
value = 4.7778

10->12

```
---------------------------------------------------------------------------------------------------

S59
Feature importance:
```

|  | importance | lifestyle |
| --- | --- | --- |
| soil/dung | 0.300024 |  |
| Sordariomycetes | 0.213944 | 0.30 animal, 0.05 fungus, 0.67 plant, 0.86 pathogen, 0.58 saprotroph, 0.02 symbiont, 0.29 soil/dung |
| pathogen | 0.200886 |  |
| Mucoromycotina | 0.134439 | 0.08 animal, 0.08 fungus, 0.08 plant, 0.25 pathogen, 0.92 saprotroph, 0.92 soil/dung |
| saprotroph | 0.109772 |  |
| Eurotiomycetes | 0.022563 | 0.66 animal, 0.01 fungus, 0.34 plant, 0.60 pathogen, 0.61 saprotroph, 0.02 symbiont, 0.47 soil/dung |
| Taphrinomycotina | 0.009557 | 0.71 animal, 0.14 fungus, 0.43 plant, 0.60 pathogen, 0.40 saprotroph, 0.14 soil/dung |
| plant | 0.008381 |  |
| animal | 0.000435 |  |

Tree


0

Sordariomycetes <= 0.5
mse = 0.8825
samples = 613
value = 0.9984

1

Mucoromycotina <= 0.5
mse = 0.3012
samples = 490
value = 0.9163

0->1


True

16

saprotroph <= 0.5
mse = 3.065
samples = 123
value = 1.3252

0->16


False

2

pathogen <= 0.5
mse = 0.2871
samples = 478
value = 0.9393

1->2


15

mse = 0.0
samples = 12
value = 0.0

1->15


3

Eurotiomycetes <= 0.5
mse = 0.1706
samples = 232
value = 0.8621

2->3


10

Taphrinomycotina <= 0.5
mse = 0.386
samples = 246
value = 1.0122

2->10


4

plant <= 0.5
mse = 0.198
samples = 184
value = 0.8261

3->4


7

plant <= 0.5
mse = 0.0417
samples = 48
value = 1.0

3->7


5

mse = 0.1924
samples = 50
value = 0.74

4->5


6

mse = 0.1963
samples = 134
value = 0.8582

4->6


8

mse = 0.0622
samples = 15
value = 1.0667

7->8


9

mse = 0.0294
samples = 33
value = 0.9697

7->9


11

Eurotiomycetes <= 0.5
mse = 0.3871
samples = 240
value = 1.0208

10->11


14

mse = 0.2222
samples = 6
value = 0.6667

10->14


12

mse = 0.5179
samples = 166
value = 0.988

11->12


13

mse = 0.0856
samples = 74
value = 1.0946

11->13


17

animal <= 0.5
mse = 0.0189
samples = 52
value = 1.0192

16->17


20

soil/dung <= 0.5
mse = 5.1771
samples = 71
value = 1.5493

16->20


18

mse = 0.0
samples = 33
value = 1.0

17->18


19

mse = 0.0499
samples = 19
value = 1.0526

17->19


21

pathogen <= 0.5
mse = 9.5432
samples = 36
value = 2.1111

20->21


24

plant <= 0.5
mse = 0.0278
samples = 35
value = 0.9714

20->24


22

mse = 0.0
samples = 8
value = 1.0

21->22


23

mse = 11.8163
samples = 28
value = 2.4286

21->23


25

mse = 0.0
samples = 20
value = 1.0

24->25


26

pathogen <= 0.5
mse = 0.0622
samples = 15
value = 0.9333

24->26


27

mse = 0.0
samples = 6
value = 1.0

26->27


28

mse = 0.0988
samples = 9
value = 0.8889

26->28

```
---------------------------------------------------------------------------------------------------

S64
Feature importance:
```

|  | importance | lifestyle |
| --- | --- | --- |
| saprotroph | 0.380932 |  |
| Eurotiomycetes | 0.295394 | 0.66 animal, 0.01 fungus, 0.34 plant, 0.60 pathogen, 0.61 saprotroph, 0.02 symbiont, 0.47 soil/dung |
| Saccharomycotina | 0.105429 | 0.49 animal, 0.33 plant, 0.47 pathogen, 0.60 saprotroph, 0.02 symbiont, 0.30 soil/dung, 0.01 water |
| pathogen | 0.063688 |  |
| Leotiomycetes | 0.057413 | 0.69 animal, 0.31 plant, 0.31 pathogen, 0.81 saprotroph, 0.06 symbiont, 0.69 soil/dung |
| soil/dung | 0.040408 |  |
| symbiont | 0.021866 |  |
| Agaricomycetes | 0.013060 | 0.01 animal, 0.97 plant, 0.15 pathogen, 0.80 saprotroph, 0.24 symbiont, 0.17 soil/dung |
| animal | 0.011277 |  |
| plant | 0.010532 |  |

Tree


0

Eurotiomycetes <= 0.5
mse = 1.4456
samples = 613
value = 0.571

1

Saccharomycotina <= 0.5
mse = 0.4624
samples = 491
value = 0.3747

0->1


True

24

saprotroph <= 0.5
mse = 4.624
samples = 122
value = 1.3607

0->24


False

2

Leotiomycetes <= 0.5
mse = 0.4547
samples = 390
value = 0.241

1->2


15

pathogen <= 0.5
mse = 0.1565
samples = 101
value = 0.8911

1->15


3

symbiont <= 0.5
mse = 0.2735
samples = 358
value = 0.176

2->3


10

soil/dung <= 0.5
mse = 1.9053
samples = 32
value = 0.9688

2->10


4

Agaricomycetes <= 0.5
mse = 0.1718
samples = 331
value = 0.136

3->4


7

Agaricomycetes <= 0.5
mse = 1.2593
samples = 27
value = 0.6667

3->7


5

mse = 0.1197
samples = 277
value = 0.1011

4->5


6

mse = 0.4009
samples = 54
value = 0.3148

4->6


8

mse = 0.41
samples = 10
value = 0.3

7->8


9

mse = 1.6332
samples = 17
value = 0.8824

7->9


11

mse = 0.36
samples = 10
value = 0.2

10->11


12

pathogen <= 0.5
mse = 2.2169
samples = 22
value = 1.3182

10->12


13

mse = 0.6875
samples = 16
value = 0.75

12->13


14

mse = 3.1389
samples = 6
value = 2.8333

12->14


16

plant <= 0.5
mse = 0.2129
samples = 53
value = 0.7736

15->16


21

saprotroph <= 0.5
mse = 0.0621
samples = 48
value = 1.0208

15->21


17

mse = 0.1928
samples = 23
value = 0.7391

16->17


18

soil/dung <= 0.5
mse = 0.2267
samples = 30
value = 0.8

16->18


19

mse = 0.2483
samples = 24
value = 0.7917

18->19


20

mse = 0.1389
samples = 6
value = 0.8333

18->20


22

mse = 0.0488
samples = 41
value = 1.0

21->22


23

mse = 0.1224
samples = 7
value = 1.1429

21->23


25

mse = 6.9475
samples = 48
value = 2.6042

24->25


26

soil/dung <= 0.5
mse = 1.4633
samples = 74
value = 0.5541

24->26


27

plant <= 0.5
mse = 4.0
samples = 17
value = 1.0

26->27


30

plant <= 0.5
mse = 0.6297
samples = 57
value = 0.4211

26->30


28

mse = 5.3542
samples = 12
value = 1.25

27->28


29

mse = 0.24
samples = 5
value = 0.4

27->29


31

animal <= 0.5
mse = 0.562
samples = 22
value = 0.2727

30->31


34

animal <= 0.5
mse = 0.6498
samples = 35
value = 0.5143

30->34


32

mse = 0.0
samples = 12
value = 0.0

31->32


33

mse = 1.04
samples = 10
value = 0.6

31->33


35

mse = 0.8533
samples = 22
value = 0.6818

34->35


36

mse = 0.1775
samples = 13
value = 0.2308

34->36

```
---------------------------------------------------------------------------------------------------

S66
Feature importance:
```

|  | importance | lifestyle |
| --- | --- | --- |
| Sordariomycetes | 0.466981 | 0.30 animal, 0.05 fungus, 0.67 plant, 0.86 pathogen, 0.58 saprotroph, 0.02 symbiont, 0.29 soil/dung |
| soil/dung | 0.254063 |  |
| pathogen | 0.073338 |  |
| Leotiomycetes | 0.072360 | 0.69 animal, 0.31 plant, 0.31 pathogen, 0.81 saprotroph, 0.06 symbiont, 0.69 soil/dung |
| saprotroph | 0.071783 |  |
| animal | 0.023124 |  |
| plant | 0.019283 |  |
| Agaricomycetes | 0.012111 | 0.01 animal, 0.97 plant, 0.15 pathogen, 0.80 saprotroph, 0.24 symbiont, 0.17 soil/dung |
| symbiont | 0.006958 |  |

Tree


0

Sordariomycetes <= 0.5
mse = 0.1544
samples = 613
value = 0.1419

1

Leotiomycetes <= 0.5
mse = 0.0602
samples = 490
value = 0.0551

0->1


True

20

soil/dung <= 0.5
mse = 0.3799
samples = 123
value = 0.4878

0->20


False

2

soil/dung <= 0.5
mse = 0.0381
samples = 458
value = 0.0349

1->2


15

animal <= 0.5
mse = 0.2881
samples = 32
value = 0.3438

1->15


3

symbiont <= 0.5
mse = 0.0188
samples = 316
value = 0.0127

2->3


8

plant <= 0.5
mse = 0.0774
samples = 142
value = 0.0845

2->8


4

mse = 0.0035
samples = 288
value = 0.0035

3->4


5

Agaricomycetes <= 0.5
mse = 0.1671
samples = 28
value = 0.1071

3->5


6

mse = 0.0
samples = 11
value = 0.0

5->6


7

mse = 0.263
samples = 17
value = 0.1765

5->7


9

animal <= 0.5
mse = 0.0149
samples = 66
value = 0.0152

8->9


12

Agaricomycetes <= 0.5
mse = 0.1238
samples = 76
value = 0.1447

8->12


10

mse = 0.0
samples = 50
value = 0.0

9->10


11

mse = 0.0586
samples = 16
value = 0.0625

9->11


13

mse = 0.1406
samples = 65
value = 0.1692

12->13


14

mse = 0.0
samples = 11
value = 0.0

12->14


16

mse = 0.09
samples = 10
value = 0.1

15->16


17

pathogen <= 0.5
mse = 0.3388
samples = 22
value = 0.4545

15->17


18

mse = 0.2148
samples = 16
value = 0.3125

17->18


19

mse = 0.4722
samples = 6
value = 0.8333

17->19


21

pathogen <= 0.5
mse = 0.3831
samples = 87
value = 0.6667

20->21


28

plant <= 0.5
mse = 0.108
samples = 36
value = 0.0556

20->28


22

mse = 0.1875
samples = 8
value = 0.25

21->22


23

saprotroph <= 0.5
mse = 0.3836
samples = 79
value = 0.7089

21->23


24

plant <= 0.5
mse = 0.4806
samples = 51
value = 0.5686

23->24


27

mse = 0.1059
samples = 28
value = 0.9643

23->27


25

mse = 0.7475
samples = 20
value = 0.55

24->25


26

mse = 0.308
samples = 31
value = 0.5806

24->26


29

mse = 0.0
samples = 21
value = 0.0

28->29


30

pathogen <= 0.5
mse = 0.2489
samples = 15
value = 0.1333

28->30


31

mse = 0.0
samples = 6
value = 0.0

30->31


32

mse = 0.3951
samples = 9
value = 0.2222

30->32

```
---------------------------------------------------------------------------------------------------
```

### Deeper trees for S10, S26 and S28¶

In [27]:

```
for protease in ['S10', 'S28']:

    Y = encoded[protease]
    
    dt = tree.DecisionTreeRegressor(min_samples_leaf=5, max_leaf_nodes=15, min_impurity_split=0.01, presort=True)
    dt.fit(X, Y)


    # Print features importance
    print(protease)
    6
    print('Feature importance:')
    feature_importances = pd.DataFrame({'importance': dt.feature_importances_}, index=features)
    feature_importances = feature_importances[feature_importances['importance'] > 0]
    feature_importances['lifestyle'] = [lifestyle_summary(data[(data['subphylum'] == i) | (data['class'] == i)])
                                        for i in feature_importances.index]
    feature_importances = feature_importances.sort_values(by='importance', ascending=False)
    display(feature_importances)

    
    # Plot tree
    dot_data = tree.export_graphviz(dt, out_file=None, feature_names=features, filled=True, rounded=True)  
    graph = pydotplus.graph_from_dot_data(dot_data)
    svg = graph.create_svg()
    display_SVG(svg)
    print('\n---------------------------------------------------------------------------------------------------\n')
```

```
S10
Feature importance:
```

|  | importance | lifestyle |
| --- | --- | --- |
| Saccharomycotina | 0.288817 | 0.49 animal, 0.33 plant, 0.47 pathogen, 0.60 saprotroph, 0.02 symbiont, 0.30 soil/dung, 0.01 water |
| Microsporidia | 0.262795 | 1.00 animal, 1.00 pathogen, 0.08 water |
| Agaricomycetes | 0.103058 | 0.01 animal, 0.97 plant, 0.15 pathogen, 0.80 saprotroph, 0.24 symbiont, 0.17 soil/dung |
| Tremellomycetes | 0.060944 | 0.66 animal, 0.03 fungus, 0.25 plant, 0.69 pathogen, 1.00 saprotroph, 0.12 soil/dung, 0.09 water |
| pathogen | 0.058222 |  |
| Eurotiomycetes | 0.036566 | 0.66 animal, 0.01 fungus, 0.34 plant, 0.60 pathogen, 0.61 saprotroph, 0.02 symbiont, 0.47 soil/dung |
| Taphrinomycotina | 0.036295 | 0.71 animal, 0.14 fungus, 0.43 plant, 0.60 pathogen, 0.40 saprotroph, 0.14 soil/dung |
| Leotiomycetes | 0.032021 | 0.69 animal, 0.31 plant, 0.31 pathogen, 0.81 saprotroph, 0.06 symbiont, 0.69 soil/dung |
| symbiont | 0.031389 |  |
| Dothideomycetes | 0.027396 | 0.03 animal, 0.95 plant, 0.59 pathogen, 0.64 saprotroph, 0.05 symbiont, 0.54 soil/dung |
| fungus | 0.023574 |  |
| Ustilaginomycotina | 0.021790 | 0.28 animal, 0.78 plant, 0.72 pathogen, 0.33 saprotroph |
| soil/dung | 0.017133 |  |

Tree


0

Saccharomycotina <= 0.5
mse = 20.5992
samples = 613
value = 7.3605

1

Microsporidia <= 0.5
mse = 20.4614
samples = 512
value = 8.1641

0->1


True

2

mse = 1.4324
samples = 101
value = 3.2871

0->2


False

3

Agaricomycetes <= 0.5
mse = 17.7995
samples = 486
value = 8.6008

1->3


4

mse = 0.0
samples = 26
value = 0.0

1->4


5

Tremellomycetes <= 0.5
mse = 13.0047
samples = 415
value = 8.0988

3->5


6

symbiont <= 0.5
mse = 35.7417
samples = 71
value = 11.5352

3->6


7

Taphrinomycotina <= 0.5
mse = 12.7734
samples = 386
value = 8.3756

5->7


8

mse = 1.4839
samples = 29
value = 4.4138

5->8


9

pathogen <= 0.5
mse = 12.3131
samples = 379
value = 8.4855

7->9


10

mse = 1.6735
samples = 7
value = 2.4286

7->10


11

Eurotiomycetes <= 0.5
mse = 10.1357
samples = 135
value = 7.3407

9->11


12

Leotiomycetes <= 0.5
mse = 12.3916
samples = 244
value = 9.1189

9->12


13

Dothideomycetes <= 0.5
mse = 10.2642
samples = 87
value = 6.3218

11->13


14

mse = 4.6107
samples = 48
value = 9.1875

11->14


19

soil/dung <= 0.5
mse = 9.1652
samples = 71
value = 5.6197

13->19


20

mse = 3.2461
samples = 16
value = 9.4375

13->20


27

mse = 15.5156
samples = 30
value = 7.1333

19->27


28

mse = 1.6157
samples = 41
value = 4.5122

19->28


15

Ustilaginomycotina <= 0.5
mse = 11.9427
samples = 234
value = 9.3162

12->15


16

mse = 0.65
samples = 10
value = 4.5

12->16


21

fungus <= 0.5
mse = 11.8245
samples = 221
value = 9.5113

15->21


22

mse = 2.3077
samples = 13
value = 6.0

15->22


23

mse = 11.2521
samples = 212
value = 9.6887

21->23


24

mse = 7.1111
samples = 9
value = 5.3333

21->24


17

pathogen <= 0.5
mse = 39.0274
samples = 54
value = 12.5185

6->17


18

mse = 12.4775
samples = 17
value = 8.4118

6->18


25

mse = 39.5133
samples = 43
value = 13.3023

17->25


26

mse = 25.3388
samples = 11
value = 9.4545

17->26

```
---------------------------------------------------------------------------------------------------

S28
Feature importance:
```

|  | importance | lifestyle |
| --- | --- | --- |
| Saccharomycotina | 0.460699 | 0.49 animal, 0.33 plant, 0.47 pathogen, 0.60 saprotroph, 0.02 symbiont, 0.30 soil/dung, 0.01 water |
| Microsporidia | 0.149587 | 1.00 animal, 1.00 pathogen, 0.08 water |
| Tremellomycetes | 0.060131 | 0.66 animal, 0.03 fungus, 0.25 plant, 0.69 pathogen, 1.00 saprotroph, 0.12 soil/dung, 0.09 water |
| Ustilaginomycotina | 0.053654 | 0.28 animal, 0.78 plant, 0.72 pathogen, 0.33 saprotroph |
| Taphrinomycotina | 0.043820 | 0.71 animal, 0.14 fungus, 0.43 plant, 0.60 pathogen, 0.40 saprotroph, 0.14 soil/dung |
| Agaricomycetes | 0.040831 | 0.01 animal, 0.97 plant, 0.15 pathogen, 0.80 saprotroph, 0.24 symbiont, 0.17 soil/dung |
| water | 0.038004 |  |
| soil/dung | 0.032943 |  |
| pathogen | 0.031354 |  |
| Eurotiomycetes | 0.028423 | 0.66 animal, 0.01 fungus, 0.34 plant, 0.60 pathogen, 0.61 saprotroph, 0.02 symbiont, 0.47 soil/dung |
| saprotroph | 0.023895 |  |
| animal | 0.018472 |  |
| Leotiomycetes | 0.018187 | 0.69 animal, 0.31 plant, 0.31 pathogen, 0.81 saprotroph, 0.06 symbiont, 0.69 soil/dung |

Tree


0

Saccharomycotina <= 0.5
mse = 3.6749
samples = 613
value = 2.0212

1

Microsporidia <= 0.5
mse = 3.435
samples = 512
value = 2.4199

0->1


True

2

mse = 0.0
samples = 101
value = 0.0

0->2


False

3

Tremellomycetes <= 0.5
mse = 3.2887
samples = 486
value = 2.5494

1->3


4

mse = 0.0
samples = 26
value = 0.0

1->4


5

Ustilaginomycotina <= 0.5
mse = 3.3417
samples = 457
value = 2.6411

3->5


6

mse = 0.2307
samples = 29
value = 1.1034

3->6


7

Taphrinomycotina <= 0.5
mse = 3.3436
samples = 439
value = 2.713

5->7


8

mse = 0.0988
samples = 18
value = 0.8889

5->8


9

water <= 0.5
mse = 3.287
samples = 432
value = 2.7546

7->9


10

mse = 0.1224
samples = 7
value = 0.1429

7->10


11

Eurotiomycetes <= 0.5
mse = 3.2357
samples = 426
value = 2.7911

9->11


12

mse = 0.1389
samples = 6
value = 0.1667

9->12


13

soil/dung <= 0.5
mse = 3.8998
samples = 304
value = 2.9605

11->13


14

mse = 1.3312
samples = 122
value = 2.3689

11->14


15

animal <= 0.5
mse = 4.3536
samples = 195
value = 3.2154

13->15


16

pathogen <= 0.5
mse = 2.7637
samples = 109
value = 2.5046

13->16


21

Agaricomycetes <= 0.5
mse = 3.8565
samples = 172
value = 3.0988

15->21


22

mse = 7.2098
samples = 23
value = 4.087

15->22


25

saprotroph <= 0.5
mse = 3.5529
samples = 114
value = 2.8684

21->25


26

mse = 4.1439
samples = 58
value = 3.5517

21->26


27

mse = 5.259
samples = 66
value = 3.2727

25->27


28

mse = 0.6732
samples = 48
value = 2.3125

25->28


17

mse = 1.5702
samples = 56
value = 1.9643

16->17


18

Agaricomycetes <= 0.5
mse = 3.3905
samples = 53
value = 3.0755

16->18


19

Leotiomycetes <= 0.5
mse = 3.2703
samples = 46
value = 3.3478

18->19


20

mse = 0.4898
samples = 7
value = 1.2857

18->20


23

mse = 3.19
samples = 40
value = 3.6

19->23


24

mse = 0.5556
samples = 6
value = 1.6667

19->24

```
---------------------------------------------------------------------------------------------------
```

## Linear models¶

Build linear models predicting number of proteases using lifestyle features (binary), taxonomy (categorical, encoded as binary), and proteome and genome sizes (continuous, scaled by their maximum).

Use stepwise elimination of insignificant features.

In [28]:

```
X = encoded[features + ['proteome_size', 'genome_size']].astype(np.float32)

# Add intercept
X['intercept'] = 1.0

# Scale proteaome and genome sizes
X[['proteome_size', 'genome_size']] /= X[['proteome_size', 'genome_size']].max()

# Save coefficients and pvalues
coefficients = pd.DataFrame(index=X.columns)
pvalues = pd.DataFrame(index=X.columns)

for protease in proteases:

    Y = encoded[protease]

    selected = list(X.columns)

    regression_model = sm.GLS(Y, X[selected])
    result = regression_model.fit()

    # Stepwise removal
    while len(selected) > 0 and result.pvalues.max() > 0.05:
        worst = result.pvalues.argmax()
        selected.remove(worst)
        regression_model = sm.GLS(Y, X[selected])
        result = regression_model.fit()

    coefficients[protease] = result.params
    pvalues[protease] = result.pvalues
```

In [29]:

```
# Save results as a single table

# Combine the tables
all_results = pd.merge(

    pd.melt(coefficients.reset_index().rename(columns={'index': 'variable'}),
            id_vars='variable', value_vars=proteases, var_name='protease', value_name='coefficient'),

    pd.melt(pvalues.reset_index().rename(columns={'index': 'variable'}),
            id_vars='variable', value_vars=proteases, var_name='protease', value_name='pvalue')

).dropna()


# Sort rows and columns
order = [i[0] for i in sorted(all_results['protease'].items(), key=lambda x: int(x[1][1:]))]
all_results = all_results.loc[order, ['protease', 'variable', 'coefficient', 'pvalue']]

all_results.to_csv('linear_models_results.tab', index=False, sep='\t')
all_results.head()
```

Out[29]:

|  | protease | variable | coefficient | pvalue |
| --- | --- | --- | --- | --- |
| 0 | S1 | animal | 2.540473 | 5.030493e-08 |
| 1 | S1 | fungus | -2.914223 | 1.798383e-02 |
| 3 | S1 | pathogen | 0.928217 | 4.389510e-02 |
| 4 | S1 | saprotroph | 0.990829 | 3.571607e-02 |
| 6 | S1 | soil/dung | 1.016954 | 2.667647e-02 |

### Plot coefficients¶

In [30]:

```
# Plot "raw" coefficients used in linear models
sns.heatmap(coefficients);
```

In [31]:

```
# Scale coefficients by the highest value of predicted variable.
# More aboundant proteases tend to have higher coefficients, so different columns are difficult to compare.

sns.heatmap(coefficients / encoded[proteases].max());
```

In [32]:

```
# Zoom-in: taxonomy
sns.heatmap(coefficients.loc[subphylum_keys+class_keys] / encoded[proteases].max());
```

In [33]:

```
# Zoom-in: lifestyle
sns.heatmap(coefficients.loc[lifestyle] / encoded[proteases].max());
```

In [34]:

```
# Scale each column to [-1, 1] range for better readability
sns.heatmap(coefficients.loc[lifestyle] / coefficients.loc[lifestyle].abs().max());
```

In [ ]:

```

```
